# Silver nanoparticles loaded with pomegranate peel extract and hyaluronic acid mediate recovery of cutaneous wounds infected with *Candida albicans*

**DOI:** 10.3389/fcimb.2024.1469493

**Published:** 2024-11-29

**Authors:** Marwa I. Abd El-Hamid, Doaa Ibrahim, Ahmed Abdelfattah-Hassan, Osama B. Mohammed, Ioan Pet, Samah S. Khalil, Sara M. El-Badry, Aya Sh. Metwally, Asmaa A. Azouz, Ahmed A. Elnegiry, Shimaa S. Elnahriry, Mirela Ahmadi, Sara T. Elazab

**Affiliations:** ^1^ Department of Microbiology, Faculty of Veterinary Medicine, Zagazig University, Zagazig, Egypt; ^2^ Department of Nutrition and Clinical Nutrition, Faculty of Veterinary Medicine, Zagazig University, Zagazig, Egypt; ^3^ Department of Anatomy and Embryology, Faculty of Veterinary Medicine, Zagazig University, Zagazig, Egypt; ^4^ Biomedical Sciences Program, University of Science and Technology, Zewail City of Science and Technology, Giza, Egypt; ^5^ Department of Zoology, College of Science, King Saudi University, Riyadh, Saudi Arabia; ^6^ Department of Biotechnology, Faculty of Bioengineering of Animals Resources, University of Life Sciences “King Mihai I” from Timisoara, Timisoara, Romania; ^7^ Department of Biochemistry & Molecular Biochemistry, Drug Information Centre, Zagazig University Hospitals, Zagazig University, Zagazig, Egypt; ^8^ Department of Animal Wealth Development, Veterinary Genetics and Genetic Engineering, Faculty of Veterinary Medicine, Zagazig University, Zagazig, Egypt; ^9^ Department of Pharmacology, Faculty of Veterinary Medicine, Aswan University, Aswan, Egypt; ^10^ Department of Pharmacology, Faculty of Veterinary Medicine, Cairo University, Giza, Egypt; ^11^ Department of Cytology and Histology, Faculty of Veterinary Medicine, Aswan University, Aswan, Egypt; ^12^ Department of Bacteriology, Mycology and Immunology, Faculty of Veterinary Medicine, University of Sadat City, Sadat City, Egypt; ^13^ Department of Pharmacology, Faculty of Veterinary Medicine, Mansoura University, Mansoura, Egypt

**Keywords:** *Candida albicans*, silver nanoparticles, pomegranate peel extract, hyaluronic acid, angiogenesis, wound closure

## Abstract

Smart innovative nanocomposites based on active ingredients and metallic nanoparticles with effective wound healing and antifungal properties are efficient in overcoming the limitations of traditional therapeutic products. Open wounds provide an ideal niche for colonization by *Candida albicans* (*C. albicans*) which poses substantial global health issues owing to delayed wound healing and disordered healing mechanisms. Therefore, proficient innovative therapies that control *C. albicans* infection and promote wound healing are of imperative importance for the management of wounds and prevention of infection and possible complications. This study aims to design a novel nanocarrier platform based on a hydrogel loaded with silver nanoparticles (AgNPs) and doped with pomegranate peel extract (PPE) and hyaluronic acid (HA), offering an unprecedented opportunity to achieve skin repair and manage *C. albicans* colonization with an efficient wound healing process. Sprague-Dawley rats (n=100) were assigned to 5 groups and infected with *C. albicans* and distributed as follows: control positive (untreated) and four cutaneous wound-healing model groups treated topically with commercial cream and PPE-HA-AgNPs at full, 50%, and 25% concentrations for 15 days, respectively. Our findings revealed that the severity of clinical signs, *C. albicans* burden, and the expression of biofilm-related genes *ALS1*, *HYR1*, and *PLB1* were diminished following treatment with PPE-HA-AgNPsIII. Notably, the formulated nanocomposite was very effective in extending the release of PPE-HA-AgNPs in infected wounds with retention percentages of 65.4% for PPE-HA-AgNPsIII. Topical administration of PPE-HA-AgNPsIII successfully alleviated the extensive inflammatory response and healed wounded skin via downregulation of tumor necrosis factor-alpha (TNF-α), interleukin-6 and IL-1 beta, and nitric oxide synthase (NOS) levels as shown by enzyme-linked immunosorbent (ELISA) and reverse transcription-quantitative polymerase chain reaction (RT-qPCR) assays. Interestingly, PPE-HA-AgNPsIII modulated angiogenic and wound healing markers as evidenced by the downregulation of MMP-9 and the upregulation of angiopoietin-1 (Ang-1), vascular endothelial growth factor (VEGF) (up to 10 days post-treatment), transforming growth factor-beta 1 (TGF-β1), *bFGF*, *EGF, Ki-*67, and collagen I and III with efficient wound closure capability. This was evidenced by the lessening of histopathological severity, which accelerated the healing of the infected skin wounds post-treatment with PPE-HA-AgNPs. Overall, our formulated PPE-HA-AgNPs provide an effective innovative therapeutic strategy for the treatment of cutaneous wounds infected with *C. albicans* with maximized wound healing efficacy, indicating their potential in clinical practice.

## Graphical Abstract

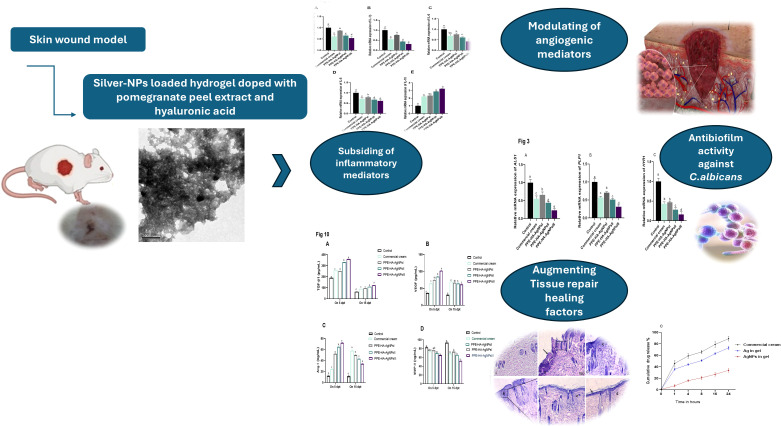

## 1 Introduction

The skin provides a natural barrier against the environment and exerts a variety of essential protective functions. Upon disruption of the skin’s integrity either by acute injuries or by chronic insults, a multi-step process is initiated, leading to at least a partial reconstruction of the wounded tissue and re-establishment of the skin’s barrier function (Okur et al., 2020). Skin wounds are any disruptions or injuries of its anatomical structure and function due to severe breakage. Wound healing remains a significant therapeutic challenge due to the complexity of the healing process. Wound healing is the result of the accumulation of processes including coagulation, inflammation, ground substance and matrix synthesis, angiogenesis, epithelialization, wound contraction, and tissue remodeling (Wang et al., 2018).

Wound infection, whether bacterial or fungal, is often the most common reason for poor wound healing. When a wound becomes infected, the degree of complication is determined by the host’s immune competence and the size of bacterial or fungal inoculums. With normal host defenses and adequate debridement, a wound may bear a level of 10^5^ pathogenic bacterial species per gram of tissue and still heal successfully (Masoko et al., 2010) but over this number, a wound may become infected. Infections with invasive fungi are still a major cause of worldwide mortality and morbidity, specifically among patients suffering from immune suppression. *Candida albicans* is a major contributor to wound infections and it is considered a global cause of opportunistic mycoses (Gil et al., 2022) with growing economic and medical importance due to extraordinary mortality rates and increased care costs and hospitalization duration (Perween et al., 2019). Moreover, the pathogenicity of *C. albicans* in open wounds is primarily related to a variety of virulence factors that help to form biofilms (Alherz et al., 2022). Up till now, treatment choices have been constrained due to the emergence of resistance to antifungal agents (Perween et al., 2019). On the other hand, various antifungal drug delivery systems to the skin are commercially offered in traditional topical forms such as creams, gels, lotions, ointments, and sprays, resulting in poor penetration (Raina et al., 2023). The problems with these topical forms are their recurrent use for many weeks until the infection signs are relieved and the existence of skin barrier functions, which hinder their delivery and consequently have low efficacy and lead to therapy failure (Firooz et al., 2015). To overcome the aforementioned issues, combining available drugs with modern technology is proposed.

Numerous efforts have been made to design novel agents with long-term efficacy against resistant *C. albicans* and no potential side effects, targeting the healing process in order to avoid the severe complications associated with chronic wounds. In this scenario, applying natural extracts as antifungal drugs is an emergent prospective field that has offered promising and interesting benefits (Nayak et al., 2017). Thus, numerous plant extracts have been investigated for wound treatment, including pomegranate peel extract (PPE), which have wound healing, antimicrobial, antioxidant, and anti-inflammatory functions that are mainly attributed to its phenolic compounds, including flavonoids (anthocyanins, catechins, and other complex flavonoids) and hydrolyzable tannins (punicalin, pedunculagin, punicalagin, gallic, and ellagic acid) (Ismail et al., 2012). These ingredients have been recognized as the fundamental bioactivity sources accounting for pomegranate’s desirable medicinal properties such as its excellent efficacy in dermal wound healing (Lukiswanto et al., 2019).

The use of nanomaterials has emerged as an enormously promising strategy to eradicate infection by virulent fungal, bacterial, and viral species (Aldakheel et al., 2023). There has been widespread employment of metallic nanoparticles to combat human pathogenic microbes in several fields, including pharmaceuticals, medicine, and biology. Metal nanoparticles have excellent antifungal activities via many mechanisms such as ion release, nitrosative and oxidative stress, enzymatic activity inhibition, cell wall and membrane damage, gene expression modulation, and mitochondrial, protein, and DNA dysfunction (Abou Hammad et al., 2020). Silver nanoparticles (AgNPs) have been broadly investigated for their prospective utilization in medicinal fields as antimicrobial agents, drug delivery systems, and nanocarriers (Lázaro-Martínez et al., 2019). AgNPs, in particular, have garnered substantial consideration for their efficacy in suppressing Gram-negative and Gram-positive bacteria, including multidrug-resistant bacterial species (Bruna et al., 2021).

Remarkably, AgNPs can attach to and invade bacterial cell membranes with subsequent destruction and leakage of cellular contents. Likewise, they can hinder essential intracellular functions such as the disruption of the respiratory chain and inhibition of cell division and DNA replication. Furthermore, they also exhibit noteworthy antimicrobial effects against antimicrobial-resistant fungal species by targeting cellular components involved in pathogenicity and drug resistance (Bruna et al., 2021). Recent technological advances in the stability and biocompatibility of AgNPs via surface alterations make them significant candidates for carrying many compounds with antifungal activity (Brown et al., 2013). The use of hydrogel scaffolds can improve the weak binding affinity of the surface of AgNPs. Thus, hydrogels can promote wound healing by maintaining equilibrium between oxygen, hydration, and chemical exchange. Hydrogels’ cross-linked three-dimensional assembly and hydrophilic polymer system permit them to play a functional role as water-sustaining scaffolds and provide a stable and efficient environment for AgNP delivery (Aldakheel et al., 2023). In addition, hyaluronic acid (HA), a natural polysaccharide, plays a fundamental role in tissue repair modulation, angiogenesis, cell motility, and signal transduction as it is a major constituent of the extracellular matrix (Dulińska-Litewka et al., 2021).

In the current study, the addition of natural compounds that possess antimicrobial and antioxidant properties to a hydrogel loaded with AgNPs may enhance their effectiveness against *C. albicans* infection, offering unique properties for biomedical applications such as their aptitude to combat fungal resistance, promote antifungal activity against planktonic and biofilm-embedded fungal species, diminish cell toxicity, and the opportunity to lessen the antimicrobial dosage (Skłodowski et al., 2023). We hypothesize that a PPE-HA-AgNP hydrogel will (a) significantly reduce *C. albicans* biofilm formation, (b) enhance wound closure, and (c) promote angiogenesis through the modulation of cytokines and growth factors and may prove to be an innovative and efficient strategy for the clinical treatment of cutaneous wounds.

## 2 Materials and methods

### 2.1 Preparation of pomegranate peel extract

Consistent with the method prescribed in a previous study (de Oliveira et al., 2013), PPE was prepared. Briefly, pomegranate peels were finely ground, dried at 50°C, and then extracted with ethanol (70%). After that, the mixture was left in the dark for 24 h at room temperature, and then purified. The prepared extract was concentrated to dryness, frozen at -70°C for 24 h for lyophilization, and then stored in a light-protected container at −20°C. A high-performance liquid chromatography (HPLC) assay was utilized to estimate the polyphenolic fractions of the prepared PPE, as shown in **Table 1**.

**Table 1:** Polyphenolic compounds of pomegranate peel extract analyzed by HPLC assay.

Analyzed extracted fractions (mg/kg)					
Rutin	2.66	Quercetrin	34.11	Vanillic	7.55
Quercetrin-3-O-glucose	1.89	Cinnamic	27.13	Ellagic	121.99
Catechol	60.15	Gallic	25.44	Apegnin	1.35
Hespirtin	5.54	Apigenin-7-glucose	7.05	Catechein	32.90
Quercetin	2.25	Naringin	9.44	Salicylic	1.31
Caffeine	12.88	Kaemp.3-(2-p-coumaroyl) glucose	8.39	Rosmarinic	13.00
Chlorogenic	18.00	p-coumaric	0.69	Acacetin7 neo hesperside	4.33
Coumarin	8.32	Ferulic	3.99	Pyrogallol	298.79
Apig.6-rhamnose galactose	7.50	Acacetin7 neo-hesperside	3.89	Apig.6-arbinose 8-galactose	3.28
Rhamentin	2.90	Benzoic	7.11	3,4,5-methoxycinnamic	1.09
Kaempferol	1.25	Luteo.7-glucose	6.23	Apig.7-O-neohesperidoside	3.15
4-Amino-benzoic	0.66	Protocatchuic	19.22	Alpha-coumaric	3.27
Iso-ferulic	0.79	Caffeic	4.60		

### 2.2 Synthesis and characterization of the nanocomposite-loaded hydrogel

**Figure 1:**
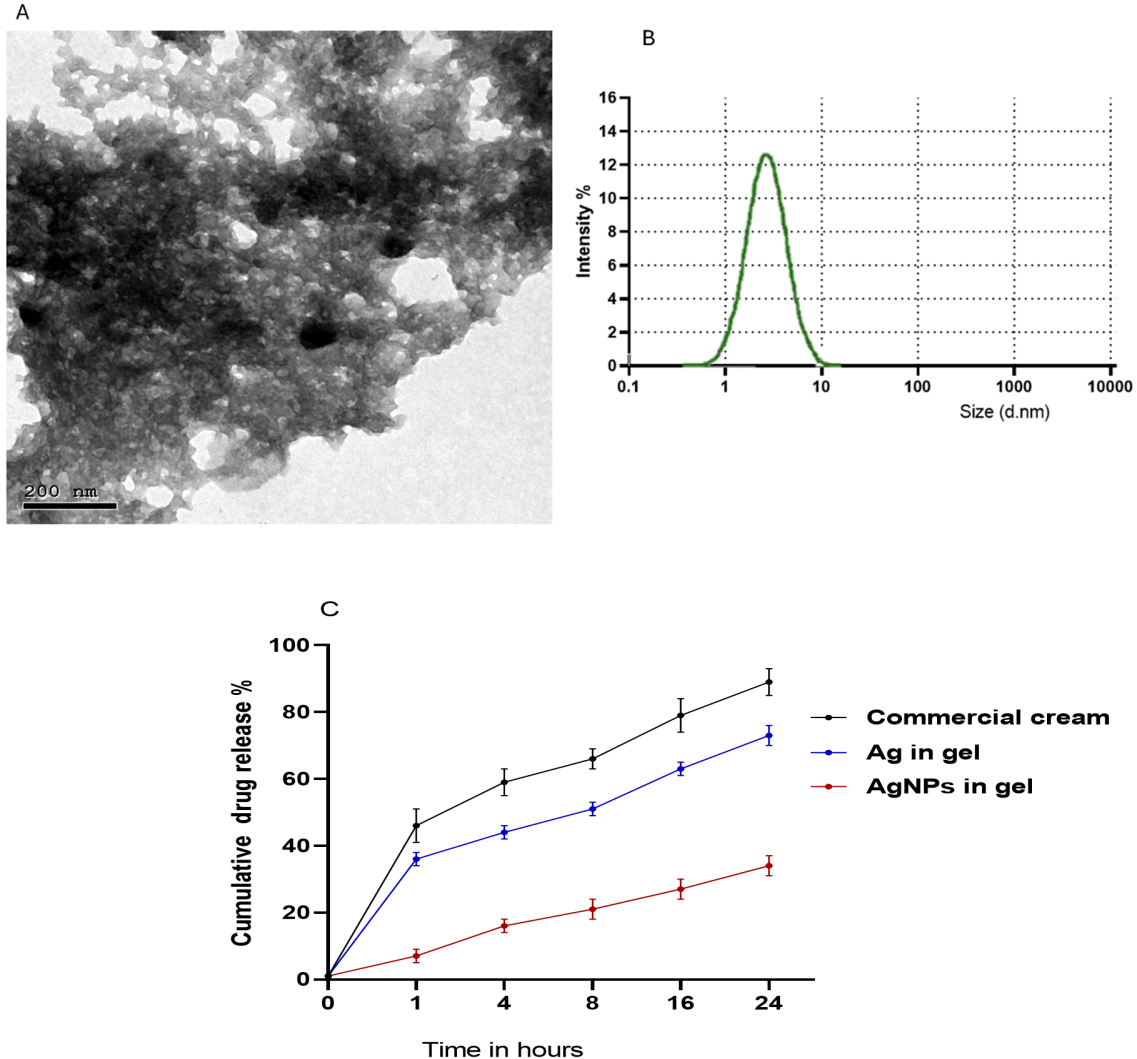
Transmission electron microscopy **(A)**, particle size distribution **(B)**, and *in vitro* release of PPE-HA-AgNP-loaded hydrogel **(C)**.

The protocols described by Gorup et al. (2011) and Das et al. (2015) were used with modifications to produce AgNPs. Briefly, 20 mL of PPE (30 mg/mL) was mixed with a solution of AgNO_3_ (0.01 M, Sigma Aldrich, St Louis, MO, USA) at a pH of 8. The reaction mixture was then left for 20 minutes at room temperature and the nanoparticle solution was centrifuged for 30 minutes at 10,000 rpm and then the collected PPE-HA-AgNP pellets were freeze-dried. The preparation of the hydrogel was carried out in line with (Ruffo et al., 2022) as carboxymethylcellulose (CMC) (Labsynth, Diadema, Brazil) was dissolved in water (2% w/v) and 20% of propylene glycol (Labsynth, Diadema, Brazil) and the mixture was stirred at room temperature for 30 minutes. Sodium hyaluronate (HA, 95%) of a low molecule weight grade (Shandong Focuschem Biotech Co, Ltda, Jinning, Shandong, China) was homogenized in purified water at 10,000 rpm for 5 min utilizing a rotor-stator (Staufen, Baden-Württemberg, Germany) to a final concentration of 0.01 g/mL. In the next step, CMC and HA were proportionally homogenized (1:1) at 10,000 rpm for 5 min using a rotor-stator (Staufen, Baden-Württemberg, Germany). Subsequently, the PPE-HA-AgNP pellets were added to the previously prepared mixture (0.01% w/w) and stirred at room temperature for 6 h. After that, citric acid was added as a crosslinking agent and the final obtained mixture was incubated at room temperature for a further 6 h. To obtain the final hydrogel loaded with PPE-HA-AgNPs, the prepared solution was freeze-dried and subsequently stored at room temperature for further characterization. The final concentrations of the ingredients in the formulated PPE-HA-AgNP-loaded hydrogel were 0.005, 0.094, and 0.34 g/mL for HA, PPE, and AgNPs, respectively. The average particle size and morphology in the synthesized PPE-HA-AgNP-loaded hydrogel were assessed using transmission electron microscopy (**Figure 1A**) at the National Centre for Radiation Research and Technology, Egypt. Furthermore, the particle size distribution of the PPE-HA-AgNP-loaded hydrogel (**Figure 1B**) was performed using dynamic light scattering (Zetasizer Nano ZS, Malvern, UK). The entrapment efficiency (EE%) of the prepared PPE-HA-AgNP-loaded hydrogel was determined according to a previously established method (Shafique et al., 2017).

### 2.3 *In vitro* release of the formulated therapeutic agents

To determine the *in vitro* release of the used therapeutic agents from the nanocomposite-loaded hydrogel, phosphate**-**buffered saline (pH=7.4) and Tween-20 (2% w/v) were added to the dissolution medium and then the nanocomposite-loaded hydrogel was dispersed in the prepared dissolution medium and magnetically stirred at 37°C (Kataoka et al., 2021). An *in vitro* release assay was performed at different intervals (1, 4, 8, 16, and 24 h, **Figure 1C**) and the amount of therapeutic agents in the examined samples were analyzed via the established HPLC assay.

### 2.4 *In vitro* anti-candidal activity of PPE-HA-AgNPs

#### 2.4.1 Fungal *C. albicans* strain and growth condition

A pathogenic clinical *C. albicans* isolate was obtained from a patient with mucocutaneous candidiasis and kept on Sabouraud dextrose agar (SDA, Oxoid, UK) slopes at 4°C. The phenotypical characteristics comprising color and morphology of the isolate were identified on HiCrome Candida differential agar medium (HiMedia, India), with the micromorphological characteristics identified on rice agar with Tween 80 (HiMedia, India), and its ability to produce germ tube was tested with biochemical tests including carbohydrate assimilation and fermentation and urease production (Kurtzman et al., 2011).

#### 2.4.2 Agar well diffusion method

The anti-candidal activity of PPE-HA-AgNPs was estimated using an agar well diffusion assay, in triplicate, as described previously (Patra and Baek, 2017). A sterile *C. albicans* culture was used to prepare a 0.5 McFarland standard suspension in 0.9% sterile saline solution with a final concentration of 10^8^ colony-forming units (CFU)/mL and the prepared fungal inoculum was spread on Sabouraud dextrose agar medium. Using a sterile cork borer, 6-mm wells were made on the surface of the agar medium. The PPE-HA-AgNP solution was prepared by dissolving the pellets in 10% dimethyl sulfoxide (DMSO; Oxoid, UK). Each well was filled with 50 μL of the prepared PPE-HA-AgNP solution which was used as a negative control and fluconazole (5 mg/mL) as a positive control. The inoculated plates were incubated at 37°C for 24 h and the zones of inhibition were then measured in millimeters.

#### 2.4.3 Broth microdilution method

A broth microdilution assay was performed, in triplicate, to determine the minimum inhibitory concentration (MIC) and minimum fungicidal concentration (MFC) of the PPE-HA-AgNPs using sterile 96-well rounded-bottom plates according to Clinical and Laboratory Standards Institute guidelines (CLSI, 2012). Briefly, Mueller–Hinton broth (Oxoid, UK) was dispensed into the microtiter plate wells and double-fold serial dilutions of PPE-HA-AgNPs with concentrations ranging from 0.625 to 10 μg/mL were made. After that, a fungal suspension prepared in brain heart infusion broth at a concentration of 5×10^5^ CFU/mL was added to each dilution. Positive (PPE-AgNP-free broth media containing fungal suspensions) and negative (fungal-free broth medium) control wells were also included. The plate was incubated at 37°C for 24 h and the culture turbidity was visually examined. The lowest concentration of PPE-HA-AgNPs (μg/mL) that had no turbidity (lack of visible fungal growth) was considered the MIC. To determine the MFC of the tested PPE-AgNPs, all fungal growth-free wells were cultured on SDA plates. The inoculated plates were incubated at 37°C for 24h and subsequently counted to determine the viable CFU/mL. The MFC that induced a fungicidal effect was considered to be the lowest concentration of PPE-HA-AgNPs that showed no visible viable fungal colonies on SDA agar plates.

### 2.5 *In vivo* wound-healing model

#### 2.5.1 Ethical statement

All animal housing and management protocols were approved by the Zagazig University’s Animal Ethics Committee. Ethical approval was attained prior to the commencement of the experimental study and all animal experiments were conducted in conformity with ethics and guidelines of the Institutional Animal Care and Use Committee of the Faculty of Veterinary Medicine, Mansura University, (MU-ACUC).

#### 2.5.2 Experimental animals and housing conditions

In total, 100 healthy male Sprague-Dawley rats (8-10 weeks old) weighing 150-200 g were obtained from the Laboratory Animals unit, Faculty of Veterinary Medicine, Zagazig University, Egypt. They were housed in separate stainless-steel cages at a temperature of 22 ± 3°C and relative humidity of 55%–65% in a 12 h light/dark cycle. All the rats were maintained under specified pathogen-free conditions and allowed a standard diet and drinking water *ad libitum*. Prior to the beginning of the experiment, the rats were allowed to acclimatize for 2 weeks under standard animal housing conditions.

#### 2.5.3 Preparation of the *C. albicans* strain and induction of fungal infection

The pathogenic clinical *C. albicans* strain cells grown on SDA medium were suspended in sterile saline to attain a target density of approximately 10^7^ CFU/mL (Barros et al., 2007). The rats were intradermally injected with 100 μL of the prepared *C. albicans* inoculum. The induced fungal infection was monitored after 3 days of inoculation in the affected skin area.

#### 2.5.4 Wound creation

The dorsal skin of rats was shaved with electrical clippers, and the exposed skin area was sterilized with 70% ethanol and allowed to dry. The rats were anesthetized with an intramuscular injection of ketamine (50 mg/kg) and xylazine (5 mg/kg). Under aseptic conditions, evenly spaced wounds were created on each animal using sterile 2 mm diameter biopsy punch equipment (Simonsen et al., 2002). Thereafter, the rats were housed individually and observed in disinfected cages to prevent infection or further wound damage.

#### 2.5.5 Experimental design

Rats were randomly allocated into five experimental groups (20 rats/group) in separate cages. After cutaneous wound creation, all rats were infected with the prepared *C. albicans* strain and distributed as follows: the control positive group with rats with untreated infected cutaneous wounds) and four wound-healing model groups with the first group comprising rats with infected cutaneous wounds that were treated with commercially available ketoconazole cream (2%) as a reference drug; the other three groups contained rats with infected cutaneous wounds that were treated with the formulated nanocomposite at three different concentrations: 25% (PPE-HA-AgNPsI), 50% (PPE-HA-AgNPsII), and 100% (PPE-HA-AgNPsIII).

The wounds were cleaned using cotton wool and all treatments were started after verification of the induced wound infection and applied topically every day for 15 days. All the animals were observed daily and examined to check for any clinical symptoms.

#### 2.5.6 Investigation of clinical lesions

The infected skin area was observed periodically to check the clinical parameters and effectiveness of the applied therapeutic agents on the 1^st^, 4^th^, and 7^th^ days each week and lesions were graded on a five-point scale (from 0 to 5) (Aggarwal et al., 2013) as follows: 0, no sign of infection; 1, slight erythematous skin; 2, redness on a well-defined area with swelling, bald patches, and scaly area; 3, large areas with redness and ulceration; 4, loss of hair and partial damage to the skin; and 5, excessive damage to the skin with complete hair loss.

#### 2.5.7 Estimation of the penetration efficacy of the used therapeutic agents

The amount of PPE-HA-AgNPs and ketoconazole cream present in the uppermost layer of the skin, stratum corneum, was estimated using the tape-stripping method as employed previously (Luengo et al., 2006). Additionally, the amount of penetrated PPE-HA-AgNPs and ketoconazole cream was determined after washing, cutting, and homogenizing the skin pieces for 2 h, followed by centrifugation for 10 min at 3,000 RCF and HPLC analysis (Minghetti et al., 2006).

#### 2.5.8 Monitoring of wound closure

The progressive decrease in the wound size and wound closure indicated by the formation of new epithelial tissue layers covering the wound were monitored periodically (every 3 days) until the 21st day of the study by tracing the wound boundaries using a transparent paper sheet and a marker. The wound healing degree was calculated as per the following formula:


$$\begin{array}{c} \text{Wound retraction }(\%)\\ =(\text{initial wound area}-\text{area of measured wound})/\text{initial wound} \end{array}$$

#### 2.5.9 Collection of skin samples

On days 5, 10, and 15 post-treatment, five rats from each group were humanely sacrificed and wound tissue specimens were excised and divided into four pieces. One wound piece was homogenized in Tris buffer saline for further assessment of some biochemical parameters including tumor necrosis factor-alpha (TNF-α), interleukin (IL)-6, IL-1 beta (IL-1β) inflammatory cytokines, nitric oxide (NO), nitric oxide synthase (NOS), C-reactive protein (CRP), and myeloperoxidase (MPO). The second wound piece was homogenized in sterile isotonic saline solution and stored at - 20°C for subsequent quantitative determination of tissue *C. albicans* burden, the third piece was kept in RNALater (Sigma Aldrich, St Louis, MO, USA) for gene expression analysis, and the last piece was fixed in neutral buffered formalin (10%) for histopathological inspection.

#### 2.5.10 Fungal burden

To assess the fungal burden on days 5, 10, and 15 post-treatment, homogenates of wound tissues were subjected to 10-fold serial dilutions and aliquots of the resultant homogenates were subsequently plated onto SDA plates. After a 48 h incubation of the inoculated SDA plates at 37°C, the number of *C. albicans* colonies was counted on each plate and expressed as CFU/g, and then log_10_ of the CFU numbers were calculated (Zhang et al., 2021). All SDA plates were carried out in duplicate and the mean fungal counts of the duplicate plates were analyzed and used for later analysis.

#### 2.5.11 Evaluation of inflammatory and wound healing-related biomarkers

All inflammatory markers were estimated on days 5 and 10 post-treatment. Rat wound tissue homogenate samples were diluted and assayed to determine the levels of TNF-α, IL-6, and IL-1β inflammatory cytokines via specific Thermo Scientific™ rat cytokine enzyme-linked immunosorbent (ELISA) kits (Cat. No. BMS622, BMS625 and BMS630, respectively). The levels of NO and NOS in the wound tissue homogenates were also estimated by colorimetric assays using QuantiChrom™ Nitric Oxide and EnzyChrom™ Nitric Oxide synthase kits, respectively. Moreover, CRP levels were measured using a commercial kit (AG723-M, Sigma-Aldrich, USA) and MPO activity was determined via an ELISA kit (E4581-100, BioVision, CA, USA). All procedures were carried out according to the manufacturer’s guidelines. Moreover, ELISA assays were utilized to estimate the concentrations of transforming growth factor-beta 1 (TGF-β1) (MultiSciences, Biotech, Co., Hangzhou, China), vascular endothelial growth factor (VEGF) (Abcam, Cambridge, USA), angiopoietin-1 (Ang-1) (Elabscience, USA) and matrix metalloproteinase-9 (MMP-9) (Elabscience, USA) on days 5 and 15 post-treatment.

#### 2.5.12 Assessment of total antioxidant capacity and lipid peroxide concentration

On day 10 post-treatment, the skin tissue homogenates were used to estimate the levels of lipid peroxides reflected by malondialdehyde (MDA) via a specific commercial colorimetric kit (Cat. No. LIP39-K01) purchased from Eagle Biosciences, Inc. (Boston, USA) and total antioxidant capacity (TAC) was calculated via a Cayman TAC assay kit (Cayman Chemical Co., Ann Arbor, USA) following manufacturer’s instructions. Furthermore, ELISA kits (MyBioSource; San Diego, USA) were utilized for the measurement of glutathione peroxidase (GSH-Px), catalase (CAT), and superoxide dismutase (SOD) activity (Cat. No. MBS028183, MBS006963 and MBS036924 respectively) according to manufacturer’s protocols. Reactive oxygen species (ROS) content in the skin tissues was determined using a specialized ELISA kit (Cat. No. MBS039665, MyBioSource; San Diego, USA) according to the manufacturer’s instructions. Moreover, hydrogen peroxide (H_2_O_2_) levels were estimated using methods detailed previously (Loreto and Velikova, 2001) and their amounts were expressed as μmoL/g of skin tissue.

#### 2.5.13 Quantification analysis using reverse transcription-quantitative polymerase chain reaction assays

Quantitative transcriptional analysis was utilized to investigate the effect of the PPE-HA-AgNPs on genes encoding inflammatory mediators, i.e., TNF-α, IL-6, IL-18, IL-1β, and IL-10, on day 5 post-treatment; on antioxidant markers, i.e., glutathione peroxidase (GSH-Px), CAT, SOD, heme oxygenase-1 (HO-1), NAD(P)H quinone oxidoreductase 1 (NQO1), and nuclear factor erythroid 2-related factor 2 (Nrf2) on day 10 post-treatment; on angiogenic and wound healing markers, i.e., matrix metalloproteinase-9 (MMP-9), collagen I and III, VEGF, Ang-1, TGF-β1, basic fibroblast growth factor (bFGF), epidermal growth factor (EGF), and antigen Kiel-67 (*Ki-*67) on days 5, 10, and 15 post-treatment; and on *C. albicans* agglutinin-like sequence 1 (*ALS1*), hyphally regulated (*HYR1*), and phospholipase B (*PLB1*) genes on day 15 post-treatment. Total *C. albicans* RNA was extracted according to the technique described in the RNeasy Mini kit (Qiagen, Germany) and then a one-step reverse transcription-quantitative polymerase chain reaction (RT-qPCR) procedure was utilized for all mRNA quantifications via a QuantiTect SYBR Green RT-PCR kit (Qiagen, Germany) on the Stratagene MX3005P (Agilent Technologies, Santa Clara, CA, USA) real-time PCR thermal cycler. All PCR reactions were conducted in three independent replicates. Melting curve analysis was subsequently carried out to confirm the presence of specific amplicons. A list of primer sets used in all RT-qPCR assays for the gene expression analyses is given in **Table 2**. The expression of *C. albicans* biofilm-associated genes was normalized against the elongation factor 1-beta (*EFB1*) housekeeping gene. Furthermore, β-actin was utilized as an internal reference gene for normalizing the expression levels of the other inflammatory, antioxidant, angiogenic, and wound healing-associated genes. The relative fold changes in target gene expression were determined using the comparative Ct method, referred to as 2^-ΔΔCt^ (Livak and Schmittgen, 2001).

**Table 2:** Primer sequences utilized for gene expression analysis via RT-qPCR assays.

Specificity/Target gene	Primer sequence (5′-3′)	Accession No./Reference
Inflammatory mediators		
*IL-1β*	F: TGACAGACCCCAAAAGATTAAGG R: CTCATCTGGACAGCCCAAGTC	NM_031512.2
*IL-6*	F: CCACCAGGAACGAAAGTCAAC R: TTGCGGAGAGAAACTTCATAGCT	NM_012589.2
*IL-18*	F: ATGGCTGCCATGTCAGAAGA R: TTGTTAAGCTTATAAATCATGCGGCCTCAGG	XM_039080945.1
*IL-10*	F: GCCCAGAAATCAAGGAGCATT R: CAGCTGTATCCAGAGGGTCTTCA	L02926.1
*TNFα*	F: CAGCCGATTTGCCATTTCA R: AGGGCTCTTGATGGCAGAGA	L19123.1
Antioxidant markers		
*CAT*	F: ACGAGATGGCACACTTTGACAG R: TGGGTTTCTCTTCTGGCTATGG	NM_012520.2
*SOD*	F: AGCTGCACCACAGCAAGCAC R: TCCACCACCCTTAGGGCTCA	NM_017051.2
*GSH-Px*	F: AAGGTGCTGCTCATTGAGAATG R: CGTCTGGACCTACCAGGAACT	NM_030826.4
*HO-1*	F: CCCAGAGGCTGTGAACTCTG R - AGGCCCAAGAAAAGAGAGCC	NM_012580.2
*Nrf2*	F: GGTTGCCCACATTCCCAAAC R - GGCTGGGAATATCCAGGGCA	NM_031789.2
*NQO1*	F: CATTCTGAAAGGCTGGTTTGA R: CTAGCTTTGATCTGGTTGTCAG	(Yeligar et al., 2010)
Angiogenic and wound healing markers		
*TGF-β1*	F: CCAGCCGCGGGACTCT R: TTCCGTTTCACCAGCTCCAT	NM_021578.2
*VEGF*	F: GATCCAGTACCCGAGCAGTCA R: TCTCCTTTCTTTTTGGTCTGCAT	NM_053549.1
*Ang-1*	F: AGGTTGGTGGTTTGATGCCT R: CGGGAACATCCCCAGATTGT	NM_017232
*MMP-9*	F: GACACCACCGAGCTATCCAC R: TTTAAACGGGCTGTTTCCCCT	(Zhao et al., 2023)
Collagen I	F: TTTGGAGAGAGCATGACCGA R: AGGGACTTCTTGAGGTTGCC	(Zhao et al., 2023)
Collagen III	F: TGCAATGTGGGACCTGGTTT R: GGGCAGTCTAGTGGCTCATC	(Zhao et al., 2023)
*Ki-67*	F: GGGTTTCCAGACACCAGACC R: CCAGGAAGACCAGTTAGAACC	NM_001271366.1
*EGF*	F: CTCAGGCCTCTGACTCCGAA R: ATGCCGACGAGTCTGAGTTG	NM_012842.1
*bFGF*	F: CGATAGAACACGGCATCAaTC R: CATCAGGCAGTTCGTAGCTC	NM_019305.2
*C. albicans* biofilm		
*ALS1*	F: CCATCACTGAAGATATCACCACA R: TGGAGCTTCTGTAGGACTGGTT	(Cheng et al., 2005)
*HYR1*	F: TTGTTTGCGTCATCAAGACTTTG R: GTCTTCATCAGCAGTAACACAACCA	(Tsang et al., 2012)
*PLB1*	F: GGTGGAGAAGATGGCCAAAA R: AGCACTTACGTTACGATGCAACA	(Nailis et al., 2010)
Housekeeping genes		
*β-actin*	F: CGCAGTTGGTTGGAGCAAA R: ACAATCAAAGTCCTCAGCCACAT	V01217.1
*EFB1*	F: TCAGATTTCTCTAAAGTCG R: TGACATCAGCTTGAGTGG	X96517

*IL*, interleukin; *TNF-α*, tumor necrosis factor-alpha; *CAT*, catalase; *SOD*, superoxide dismutase; *GSH-Px*, glutathione peroxidase; *HO-1*, heme oxygenase-1; *Nrf2*, nuclear factor erythroid 2-related factor 2; *NQO1*, NAD(P)H quinone oxidoreductase 1; *TGF-β1*, transforming growth factor beta 1; *VEGF*, vascular endothelial growth factor; *Ang-1*, angiopoietin-1; *MMP-9*, matrix metalloproteinase-9; *Ki-67*, Kiel-67; *EGF*, epidermal growth factor; *bFGF*, basic fibroblast growth factor; *ALS1*, *C. albicans* agglutinin-like sequence 1; *HYR1*, hyphally regulated gene; *PLB1*, phospholipase B; *EFB1*, elongation factor 1-beta.

#### 2.5.14 Histopathological analysis

On days 5 and 15 post-treatment, the rat skin tissues in all the experimental groups were fixed in neutral buffered formalin solution (10%) for 24 h, and then dehydrated in grade ethanol, cleaned in xylene, embedded in paraffin, and cut using a Leica microtome. After that, prepared skin tissue sections (5-μm) were subjected to staining with hematoxylin and eosin (H&E) and examined under light microscopy (Bancroft and Gamble, 2008).

### 2.6 Statistical analysis

Statistical assessment of all measured parameters was performed using one-way analysis of variance (ANOVA) via SPSS statistical software version 20 (IBM Corp., USA SPSS^®^ program version 22 (SPSS Inc., Chicago, IL, USA) with a sample size of n=5/group. Validating the statistical methods was done via homogeneity and variance normality using Levene and Shapiro–Wilk tests, respectively. Variations among the studied experimental groups were examined using Tukey’s *post-hoc* test. All data were presented as means ± standard errors with statistically significant values at *p* < 0.05. All the graphs were created using GraphPad Prism (Version 8, GraphPad Software Inc.). The fold change was assessed by the following equation: (B–A)/A, where the least value is A and the maximum value is B. Relative fold changes in the expression of specific genes were calculated by the 2^-ΔΔCt^ method (Livak and Schmittgen, 2001).

## 3 Results

### 3.1 *In vitro* anti-candidal activity of the PPE-HA-AgNPs

Regarding the susceptibility of the *C. albicans* strain to the PPE-HA-AgNPs, the investigated strain was highly susceptible to the formulated nanocomposite, with a recorded inhibition zone diameter of 34 mm as estimated via an agar well diffusion assay and the corresponding MIC and MFC values of 1.25 and 2.5 μg/mL, respectively, as determined by the broth microdilution method.

### 3.2 Clinical observation

The efficacy of the PPE-HA-AgNP-loaded hydrogel on lesion severity was described and scored accordingly, as shown in **Table 3**. It was observed that the infected and untreated rats displayed no signs of improvement with clinical findings of excessive hair loss, redness, and damage to the skin. Furthermore, the group treated with PPE-HA-AgNPsI showed little improvement with moderate areas of redness and ulceration and at the end of treatment period, the infected area in this group was not completely cured. The traditionally and PPE-HA-AgNPsII-treated groups showed slight redness in the defined areas with mild swelling, bald patches, and scaly areas. However, treatment with the PPE-HA-AgNPs at full concentration achieved the maximum curing capability for *C. albicans* infection compared with other treated groups.

**Table 3:** Effect of PPE-HA-AgNP-loaded hydrogel at different concentrations on lesion severity.

Group	Lesion	Score
Control	Excessive damage to the skin with complete hair loss	5
Commercial cream	Slight redness, mild swelling, bald patches, and scaly areas	2
PPE-HA-AgNPsI	Large areas with redness and ulceration	3
PPE-HA-AgNPsII	Redness on a well-defined area with swelling, bald patches, and scaly areas	2
PPE-HA-AgNPsIII	Slightly erythematous skin	1

Control: the rats in this group had an experimental cutaneous wound with subsequent *C. albicans* strain infection and received no treatment; commercial cream: rats in this group had an experimental cutaneous wound with subsequent *C. albicans* infection and received a commercially available ketoconazole cream (2%) as a reference drug; PPE-HA-AgNPsI: the rats in this group had an experimental cutaneous wound with subsequent *C. albicans* infection and received a PPE-HA-AgNP formulated nanocomposite at 25% concentration; PPE-HA-AgNPsII: the rats in this group had an experimental cutaneous wound with subsequent *C. albicans* infection and received a PPE-HA-AgNP formulated nanocomposite at 50% concertation; PPE-HA-AgNPsIII: the rats in this group had an experimental cutaneous wound with subsequent *C. albicans* infection and received a PPE-HA-AgNP formulated nanocomposite at full concentration.

### 3.3 Fungal burden and expression of *C. albicans* biofilm-associated genes

**Figure 2:**
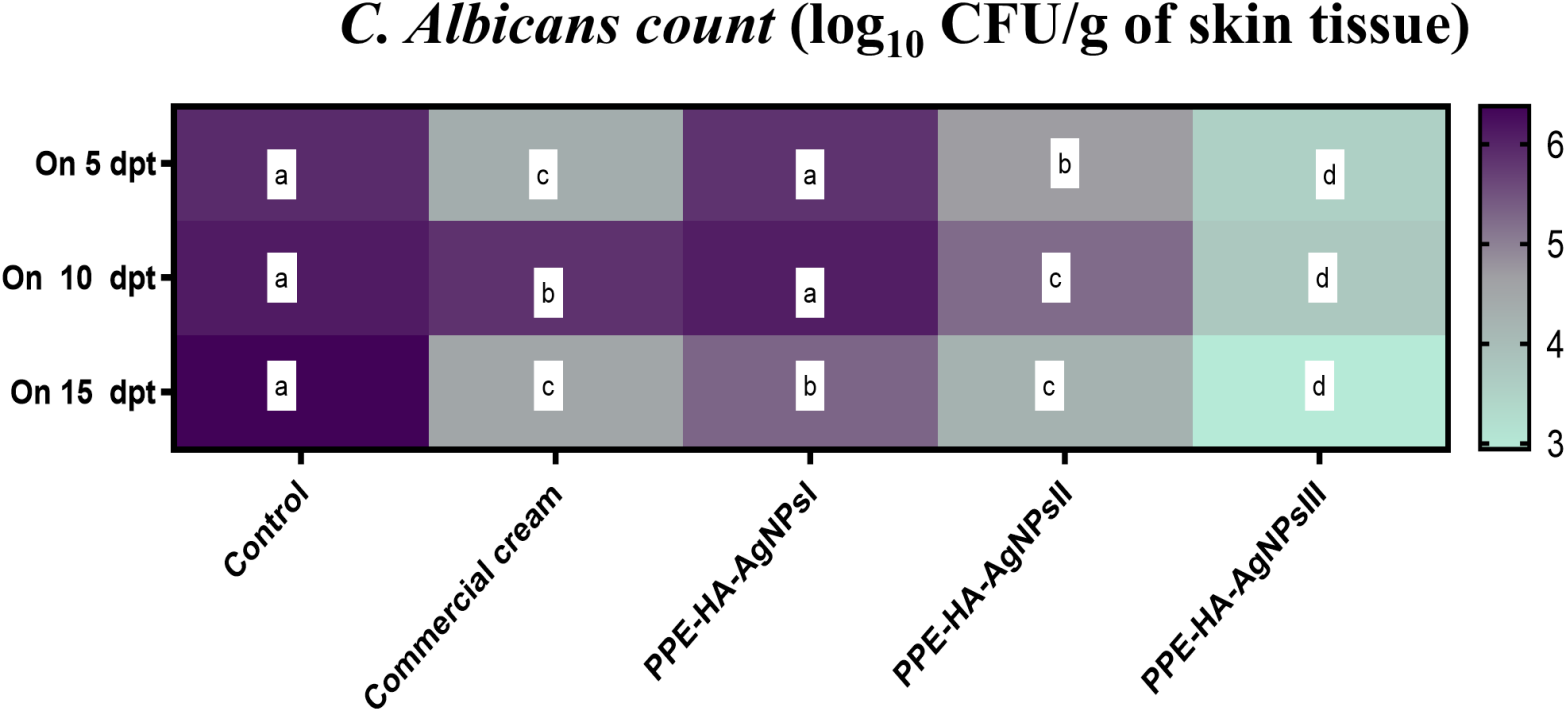
Heat map illustrating the quantification of *C. albicans* burden at three intervals [5,10 and 15 days post-treatment (dpt)] in response to the therapeutic effect of a hydrogel loaded with pomegranate peel extract-hyaluronic acid-silver nanoparticles (PPE-HA-AgNPs) at different concentrations for 15 days in cutaneous wounds in rat experimentally infected with *C. albicans.* Control: the rats in this group had an experimental cutaneous wound with subsequent *C. albicans* strain infection and received no treatment, commercial cream: rats in this group had an experimental cutaneous wound with subsequent *C. albicans* infection and received a commercially available ketoconazole cream (2%) as a reference drug, PPE-HA-AgNPsI: the rats in this group had an experimental cutaneous wound with subsequent *C. albicans* infection and received a PPE-HA-AgNPs formulated nanocomposite at 25% concentration, PPE-HA-AgNPsII: the rats in this group had an experimental cutaneous wound with subsequent *C. albicans* infection and received a PPE-HA-AgNPs formulated nanocomposite at 50% concertation, PPE-HA-AgNPsIII: the rats in this group had an experimental cutaneous wound with subsequent *C. albicans* infection and received a PPE-HA-AgNPs formulated nanocomposite at full concentration. ^a–d^: Mean values with distinct letters in the same row changed significantly (*p* < 0.05).

In relation to the outcomes of quantitative analysis of *C. albicans* in skin tissues, lower *C. albicans* populations were found in all the PPE-HA-AgNP-treated groups in a dose-dependent manner compared to the control untreated group and these counts steadily diminished over time. Notably, significantly lower (*p* < 0.05) *C. albicans* populations were detected in the skin tissues of the rats treated with PPE-HA-AgNPsIII compared with the control untreated group (**Figure 2**). The most relevant finding was reported post-treatment with PPE-HA-AgNPsIII, where it was found to have significantly reduced *C. albicans* counts by 2.95 log_10_ CFU/g on day 15 following treatment.

**Figure 3:**
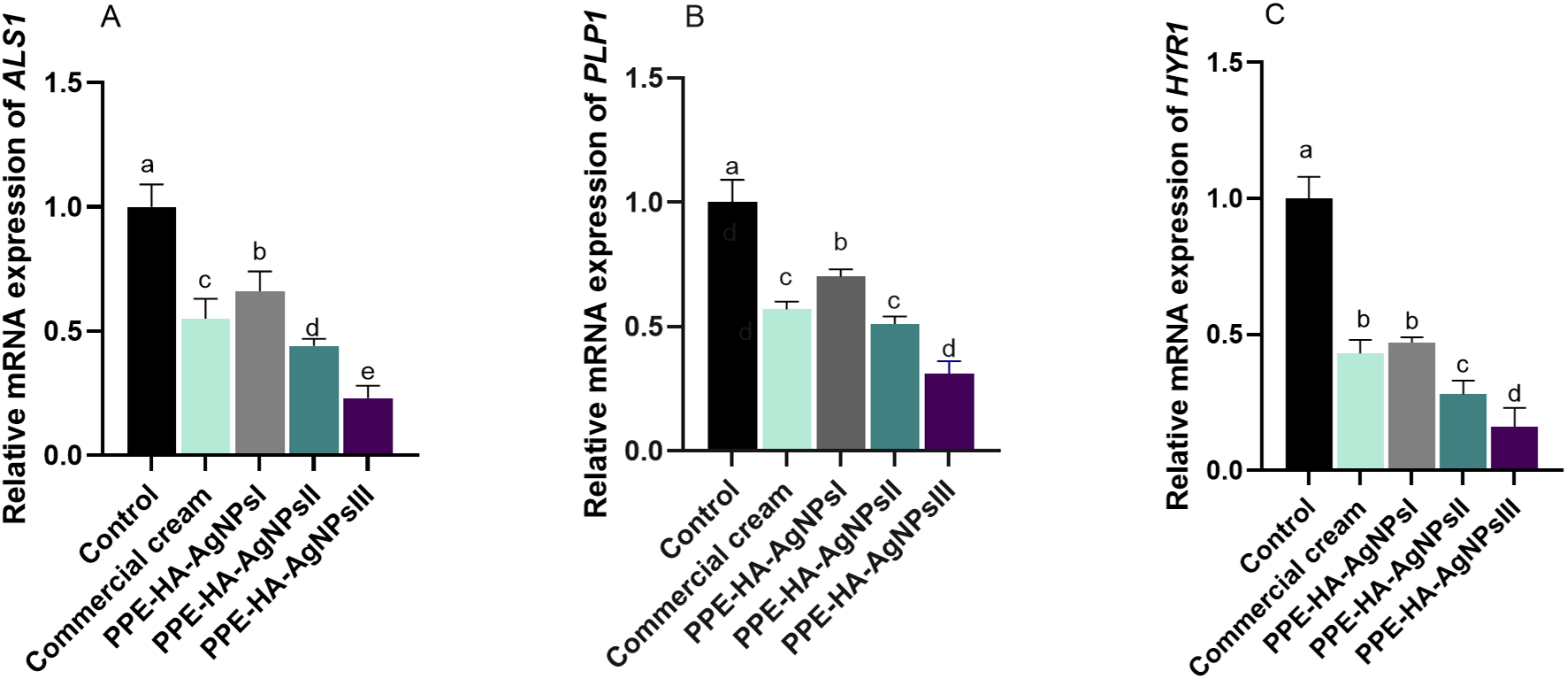
Relative mRNA expression of *C. albicans ALS1* [agglutinin-like sequence 1, **(A)**], *PLB1* [phospholipase B, **(B)**], and *HYR1* [hyphally regulated, **(C)**] biofilm-related genes on day 15 post-treatment in response to therapeutic efficacy of hydrogel loaded with pomegranate peel extract-hyaluronic acid-silver nanoparticles (PPE-HA-AgNPs) by different concentrations for 15 days in rat cutaneous wound experimentally infected with *C. albicans*. Control: *C. albicans* infected and non-treated rats, commercial cream: ketoconazole cream (2%), PEE-HA-AgNPsI: 25% concentration of formulated nanocomposite, PPE-HA-AgNPsII: 50% concentration of formulated nanocomposite, PPE-HA-AgNPsIII: full concentration of formulated nanocomposite. ^a–e^Mean values with distinct letters in the same column changed significantly (*p* < 0.05).

The relative mRNA expression of *C. albicans* biofilm-related genes tended to be downregulated on day 15 after treatment with PPE-HA-AgNPs in a dose-dependent manner when compared to the control untreated group (**Figure 3**). Notably, lower expression levels of the *ALS1* gene were observed in the group treated with PPE-HA-AgNPsIII (0.23- fold change), followed by the PPE-HA-AgNPsII group (0.44- fold change). Moreover, the *HYR1* gene was downregulated in these groups (0.16 and 0.28-fold changes, respectively). Furthermore, PPE-HA-AgNPsIII, PPE-HA-AgNPsII, and the topical treatment led to downregulation of the *PLB1* gene (0.31, 0.51, and 0.57-fold changes, respectively) unlike the control untreated group. The most pronounced reduction (*p* < 0.05) in *ALS1*, *HYR1*, and *PLB1* expression levels was detected in the skin tissues of the rats treated with PPE-HA-AgNPsIII (0.23, 0.16, and 0.31-fold changes, respectively).

### 3.4 Penetration and retention efficacy of the PPE-HA-AgNPs

**Figure 4:**
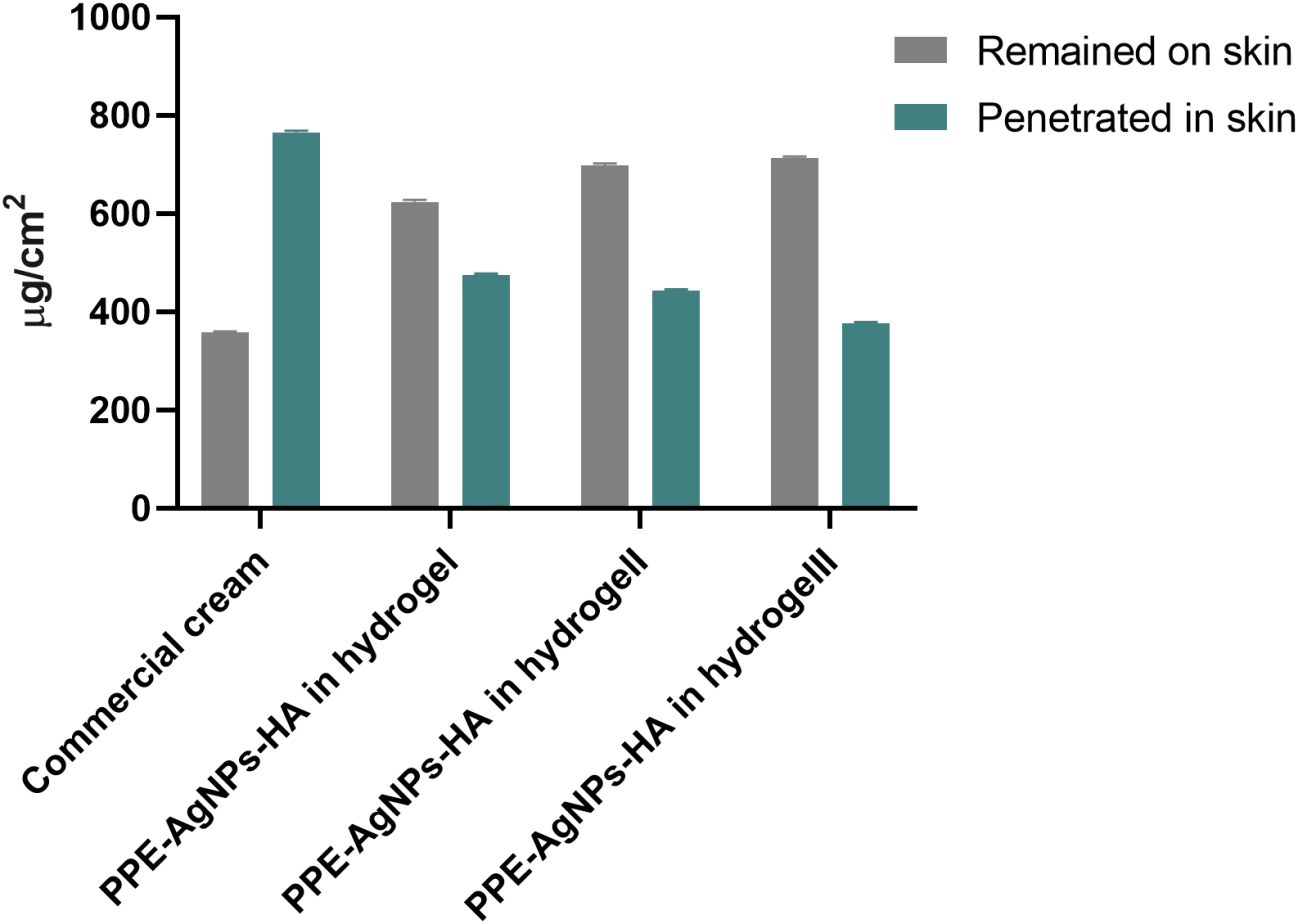
Effects of therapeutic concentrations of pomegranate peel extract-hyaluronic acid-silver nanoparticles (PPE-HA-AgNPs) on cutaneous wounds in experimental rats. Commercial cream: ketoconazole cream (2%), PEE-HA-AgNPsI: 25% concentration of the formulated nanocomposite, PPE-HA-AgNPsII: 50% concentration of the formulated nanocomposite, PPE-HA-AgNPsIII: full concentration of the formulated nanocomposite.

As shown in **Figure 4**, a small amount of the commercial cream (358 μg/cm2, 31.87%) was retained on the skin surface of rats after its topical application. Remarkably, the full concentration of PPE-HA-AgNPs showed more retention on the skin surface (713 μg/cm2, 65.4%) compared with the other concentrations (PPE-HA-AgNPsII: 698 μg/cm2, 61.2%; and PPE-HA-AgNPsI: 624 μg/cm2, 56.7%). Moreover, the retention analysis performed to evaluate the amount of therapeutic agent retained in the skin after 24 h demonstrated that the penetration of the commercial cream was much higher (68.12%) than PPE-HA-AgNPsIII (34.52%).

### 3.5 Wound closure as an endpoint of PPE-HA-AgNP treatment efficacy

**Figure 5:**
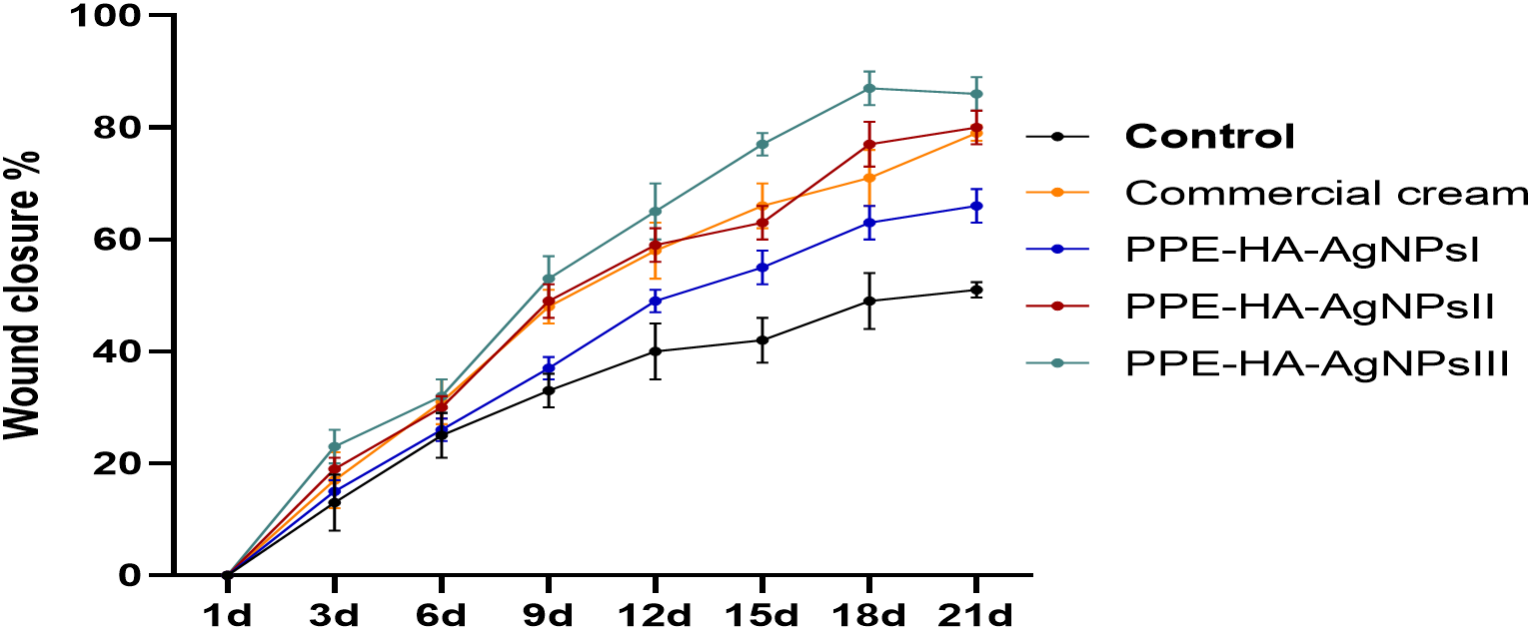
The percentage of wound closure measured at 1, 3, 6, 9, 12, 15, 18, and 21 days after wound creation. Commercial cream: ketoconazole cream (2%), PEE-HA-AgNPsI: 25% concentration of the formulated nanocomposite, PPE-HA-AgNPsII: 50% concentration of the formulated nanocomposite, PPE-HA-AgNPsIII: full concentration of the formulated nanocomposite.

As illustrated in **Figure 5**, the group treated with PPE-HA-AgNPsIII displayed significant *(p* < 0.05) wound retraction compared to the other treated groups at all the investigated intervals. Moreover, the wound closure rate in the PPE-HA-AgNPsIII-treated group on day 21 of the study was over 85%, while that in the groups treated with PPE-HA-AgNPsII and the commercial cream was 80% and 79%, respectively.

### 3.6 Investigating the healing and therapeutic impact of PPE-HA-AgNPs on inflammatory markers

The effect of the PPE-HA-AgNP-loaded hydrogel on the inflammatory markers, measured using ELISA assays, is shown in **Table 4**. Notably, TNF-α and IL-6 levels were significantly decreased following treatment with PPE-HA-AgNPs, in a dose-dependent manner, unlike the ketoconazole cream-treated group, especially on day 10 after treatment. On day 5 following treatment, IL-1β level revealed no significant variations (*p* > 0.05) among the ketoconazole cream, PPE-HA-AgNPsII, and PPE-HA-AgNPsIII-treated groups; meanwhile, the most significant reduction in IL-1β level was detected in the PPE-HA-AgNPsIII-treated group on day 10 after treatment.

**Table 4:** Inflammatory markers in response to therapeutic effects of a hydrogel loaded with pomegranate peel extract-hyaluronic acid-silver nanoparticles at different concentrations in cutaneous wounds in rats experimentally infected with *C. albicans*.

Parameter	Experimental groups					*P* value	SEM
On 5 dpt	Control	Commercial cream	PPE-HA-AgNPsI	PPE-HA-AgNPsII	PPE-HA-AgNPsIII		
**TNF-α, pg/μL**	327.33^a^	171.67^c^	212.33^b^	173.00^c^	153.08^d^	0.02	1.2
**IL-6, pg/μL**	179.10^a^	159.00^b^	137.07^c^	97.67^d^	71.87^e^	< 0.001	1.2
**IL-1β, pg/μL**	194.00^a^	131.00^c^	149.00^b^	133.33^c^	127.33^c^	0.02	2.6
**NO (µmol/ L)**	4.93^a^	4.83^a^	3.80^b^	3.10^c^	2.77^c^	< 0.001	0.06
**NOS (µmol/ L)**	1.87^a^	1.46^ab^	1.40^ab^	1.37^ab^	1.20^b^	0.04	0.05
**CRP (mg/ L)**	14.50^a^	11.33^ab^	11.43^ab^	8.57^bc^	6.90^c^	0.001	0.23
**MPO (µmol/L)**	12.53^d^	37.00^b^	32.23^c^	44.57^a^	45.53^a^	< 0.001	0.34
On 10 dpt							
**TNF-α, pg/μL**	374.33^a^	138.00^c^	164.00^b^	128.00^c^	109.97^d^	0.03	3.2
**IL-6, pg/μL**	202.77^a^	147.00^b^	125.33^c^	95.90^d^	70.50^e^	< 0.001	1.8
**IL-1β, pg/μL**	241.00^a^	131.67^b^	134.00^b^	121.33^bc^	110.67^c^	0.01	2.7
**NO (µmol/ L)**	4.17^a^	3.57^ab^	3.23^bc^	2.77^cd^	2.30^d^	< 0.001	0.1
**NOS (µmol/ L)**	1.47^a^	1.33^ab^	1.10^ab^	1.10^ab^	0.9^b^	0.03	0.04
**CRP (mg/ L)**	16.80^a^	8.97^b^	9.43^b^	6.77^b^	3.37^c^	< 0.001	0.17
**MPO (µmol/L)**	9.03^c^	31.33^a^	27.83^b^	34.27^a^	33.50^a^	< 0.001	0.15

dpt, days post-treatment; TNF-α, tumor necrosis factor-alpha; IL-6, interleukin-6; IL-1β, interleukin-1 beta; NO, nitric oxide; NOS, nitric oxide synthase; CRP, C-reactive protein; MPO, myeloperoxidase. Control: the rats in this group had an experimental cutaneous wound with subsequent *C. albicans* strain infection and received no treatment, commercial cream: rats in this group had an experimental cutaneous wound with subsequent *C. albicans* infection and received a commercially available ketoconazole cream (2%) as a reference drug, PPE-HA-AgNPsI: the rats in this group had an experimental cutaneous wound with subsequent *C. albicans* infection and received a PPE-HA-AgNP formulated nanocomposite at 25% concentration, PPE-HA-AgNPsII: the rats in this group had an experimental cutaneous wound with subsequent *C. albicans* infection and received a PPE-HA-AgNP formulated nanocomposite at 50% concertation, PPE-HA-AgNPsIII: the rats in this group had an experimental cutaneous wound with subsequent *C. albicans* infection and received a PPE-HA-AgNP formulated nanocomposite at full concentration. ^a–e^Mean values with distinct letters in the same row changed significantly (*p* < 0.05).

On day 5 post-treatment, NOS levels did not vary significantly (*p* > 0.05) among all the treated groups, while CRP and NO levels were significantly (*p* < 0.05) lower in PPE-HA-AgNPsII and PPE-HA-AgNPsIII groups, followed by the ketoconazole cream-treated group, compared to the control untreated group. On day 10 after treatment, NO, NOS, and CRP activity in the wound tissues were significantly (*p* < 0.05) diminished in the group treated with the higher concentration of PPE-HA-AgNPs compared with the ketoconazole cream-treated group. On days 5 and 10 following treatment, the PPE-HA-AgNPsII and PPE-HA-AgNPsIII-treated groups exhibited lower MPO in wound tissues compared with the other two (ketoconazole cream and PPE-HA-AgNPsI) treated groups.

**Figure 6:**
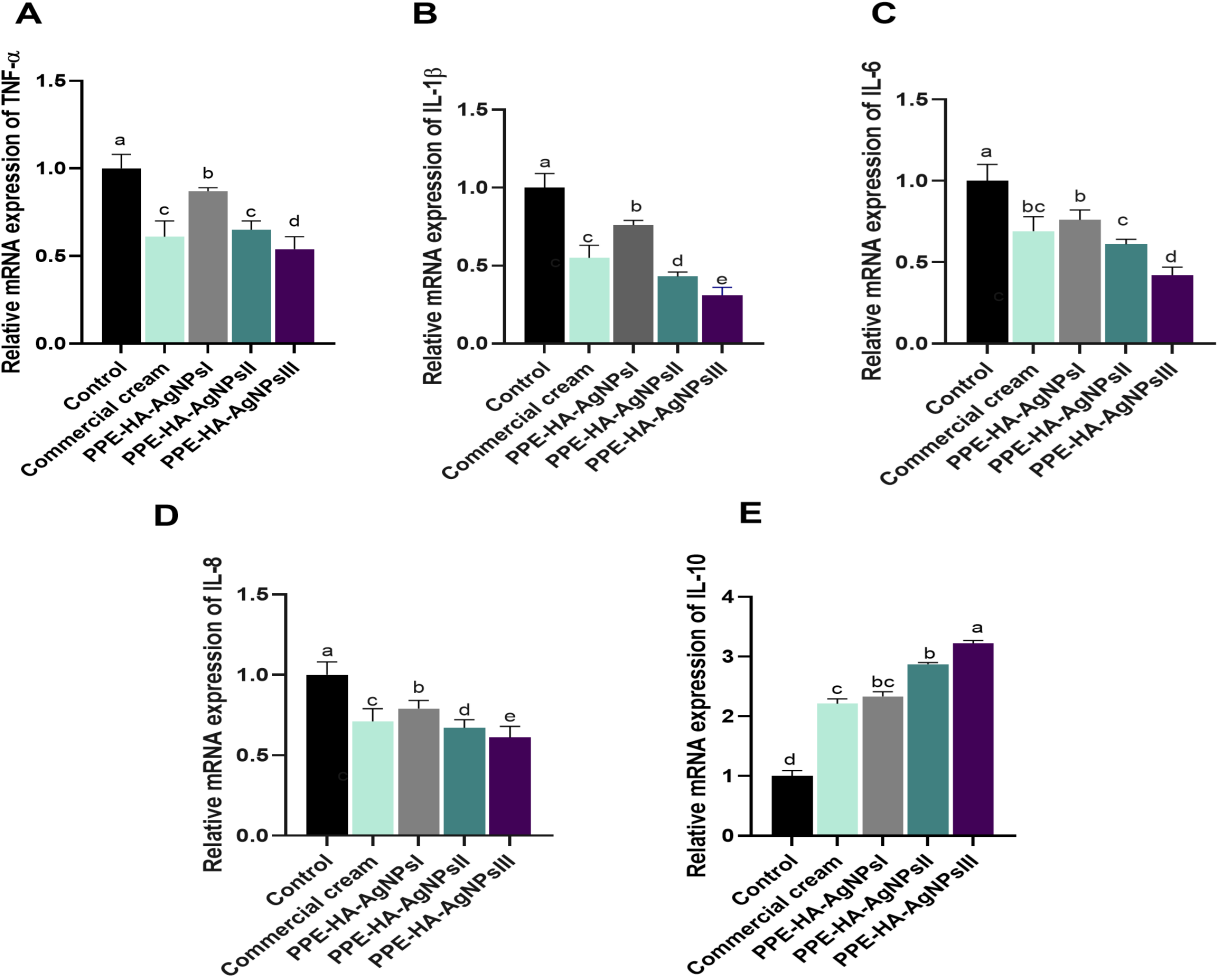
Relative mRNA expression of genes encoding cytokines' mediators: *TNF-α*, tumor necrosis factor-alpha **(A)**; *IL-1β*, interleukin-1 beta **(B)**; *IL-6*, interleukin-6 **(C)**; *IL-18*, interleukin-8 **(D)**; and *IL-10*, interleukin-10 **(E)** on day 5 post-treatment in response to the therapeutic effect of a hydrogel loaded with pomegranate peel extract-hyaluronic acid-silver nanoparticles (PPE-HA-AgNPs) at different concentrations for 15 days in cutaneous wounds in rats experimentally infected with *C*. *albicans.* Control: *C. albicans* infected and non-treated rats, commercial cream: ketoconazole cream (2%), PEE-HA-AgNPsI: 25% concentration of the formulated nanocomposite, PPE-HA-AgNPsII: 50% concentration of the formulated nanocomposite, PPE-HA-AgNPsIII: full concentration of the formulated nanocomposite. ^a–e^Mean values with distinct letters in the same column changed significantly (*p* < 0.05).

Regarding the quantitative evaluation of inflammatory markers by RT-qPCR assays on day 5 after treatment (**Figure 6**), *TNF-α* levels were significantly (*p* < 0.05) downregulated in the PPE-HA-AgNPsIII group, followed by the ketoconazole cream and PPE-HA-AgNPsII-treated groups compared with the control untreated group. Notably, the expression levels of the *IL-6*, *IL-18*, and *IL-1β* genes in the rats following topical treatment with the PPE-HA-AgNPs were dose-dependent, where the full concentration of PPE-HA-AgNPs caused significant (*p* < 0.05) reductions in their expression (0.42, 0.61 and 0.31-fold changes, respectively). Inversely, treatment with PPE-HA-AgNPs at the higher concentration caused an increase in *IL-10* expression (3.22-fold change) compared with the control untreated group.

### 3.7 Investigating the healing and therapeutic impact of PPE-HA-AgNPs on oxidative and antioxidant-related markers

**Figure 7:**
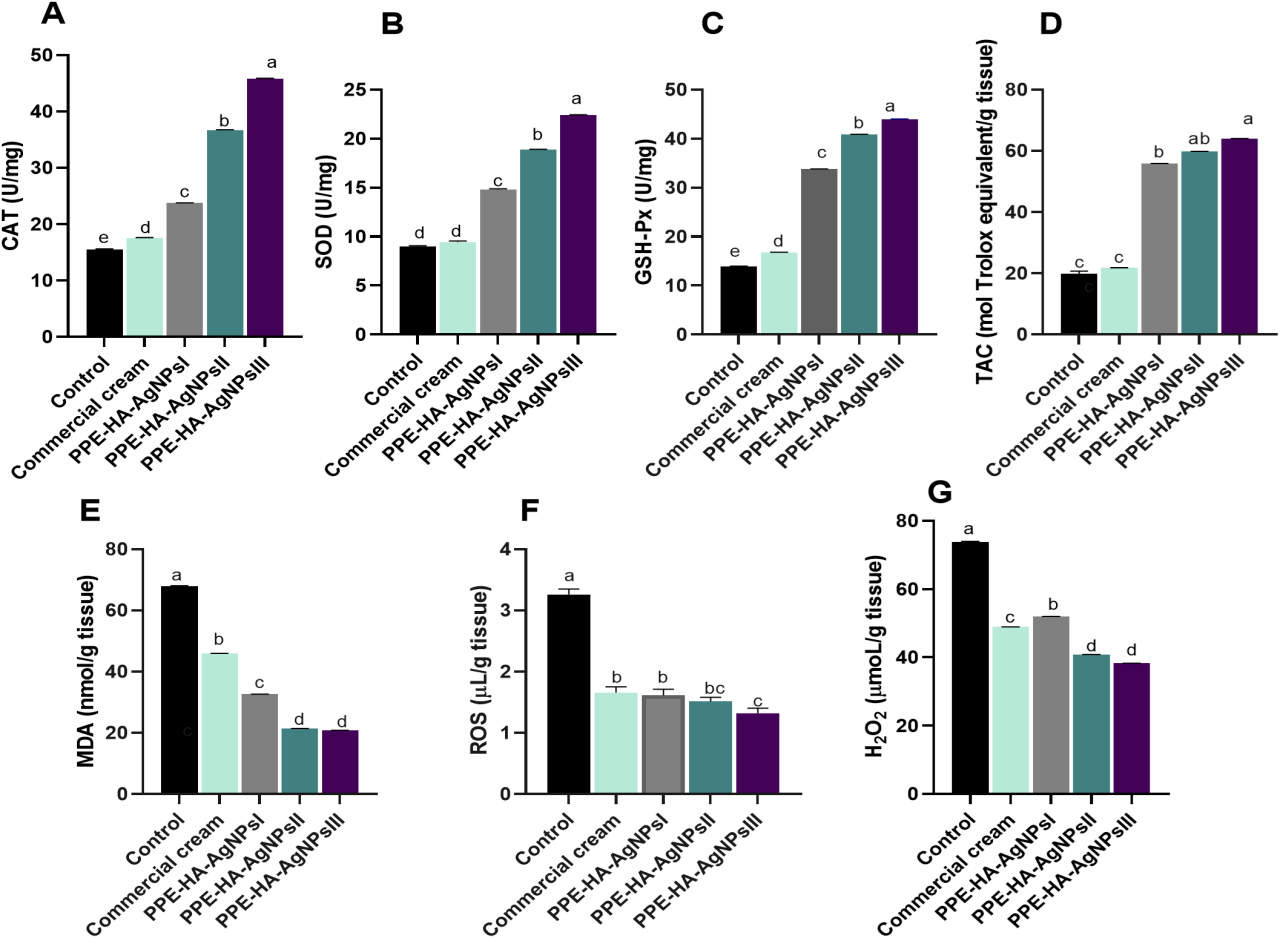
The activity of antioxidant enzymes: CAT [catalase, **(A)**], SOD [superoxide dismutase, **(B)**] glutathione peroxidase [GSH-Px, **(C)**], TAC [total antioxidant capacity, **(D)**], and lipid peroxidation biomarker [MDA, **(E)**]; and oxidative stress-related biomarkers: ROS [reactive oxygen species, **(F)**] and H_2_O_2_ [hydrogen peroxide, **(G)**] on day 10 post-treatment in response to the therapeutic effects of a hydrogel loaded with pomegranate peel extract-hyaluronic acid-silver nanoparticles (PPE-HA-AgNPs) at different concentrations for 15 days in cutaneous wounds in rats experimentally infected with *C*. *albicans.* Control: *C. albicans* infected and non-treated rats, commercial cream: ketoconazole cream (2%), PEE-HA-AgNPsI: 25% concentration of the formulated nanocomposite, PPE-HA-AgNPsII: 50% concentration of the formulated nanocomposite, PPE-HA-AgNPsIII: full concentration of the formulated nanocomposite. ^a–e^Mean values with distinct letters in the same column changed significantly (*p* < 0.05).

As illustrated in **Figure 7**, the group treated with PPE-HA-AgNPsIII, followed by PPE-HA-AgNPsII and then the PPE-HA-AgNPsI-treated groups, presented higher antioxidant enzyme activity unlike the topically treated one. The levels of CAT, SOD, and GSH-Px were significantly increased (*p* < 0.05) in the PPE-HA-AgNPsIII-treated group compared with the control group. Additionally, the skin tissue of the rats treated with PPE-HA-AgNPs at various concentrations had higher TAC and lower MDA contents compared with the control untreated rats. Furthermore, the control untreated group had higher contents of ROS and H_2_O_2_ compared with the groups treated with PPE-HA-AgNPsII and PPE-HA-AgNPsIII (*p* < 0.05).

**Figure 8:**
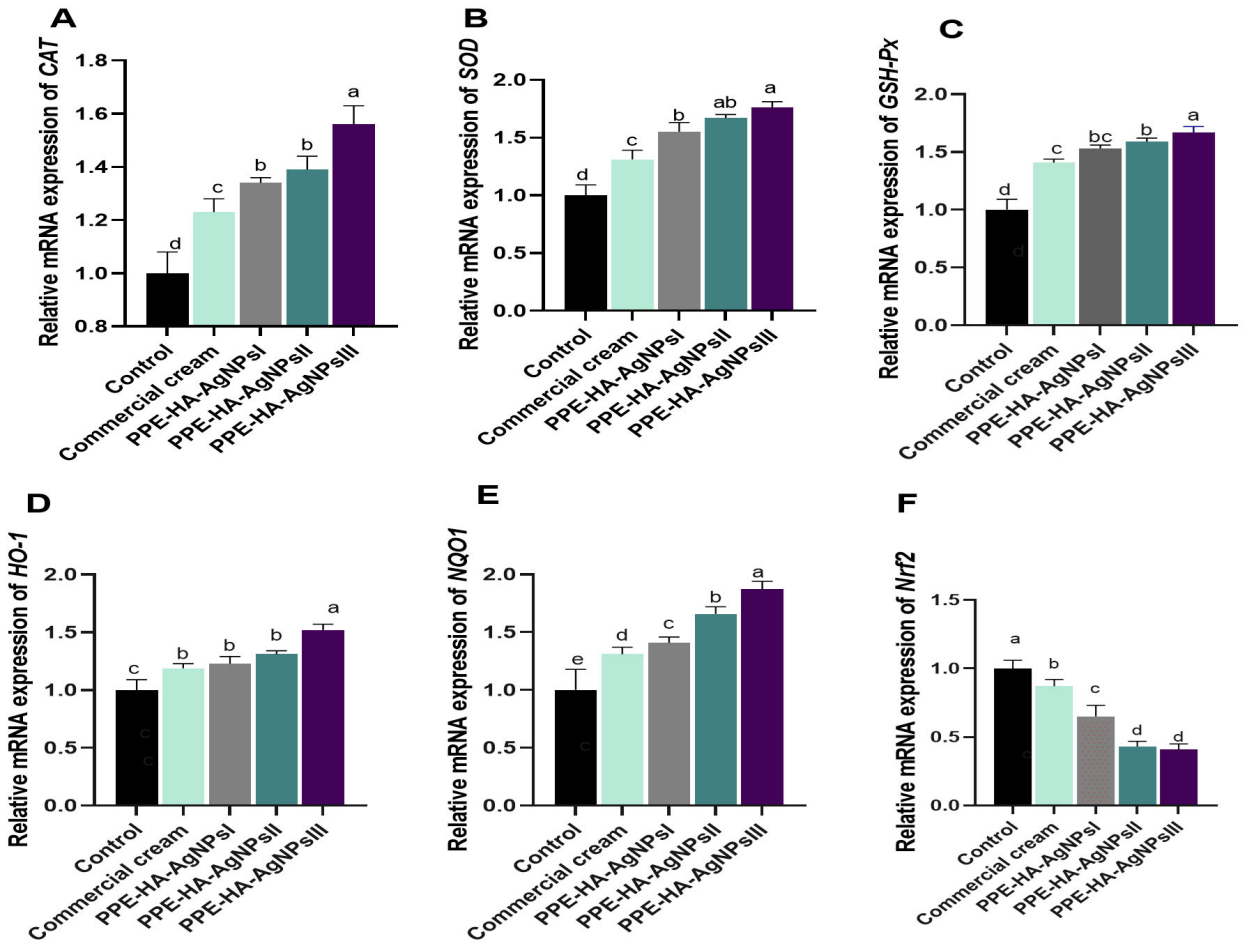
Relative mRNA expression of genes encoding antioxidants response: *CAT* [catalase, **(A)**], *SOD* [superoxide dismutase, **(B)**], *GSH-Px* [glutathione peroxidase, **(C)**], *HO-1* [heme oxygenase-1, **(D)**], *NQO1* [NAD(P)H quinone oxidoreductase 1, **(E)**], and *Nrf2* [nuclear factor erythroid 2-related factor 2, **(G)**] on day 10 post-treatment in response to the therapeutic effects of a hydrogel loaded with pomegranate peel extract-hyaluronic acid-silver nanoparticles (PPE-HA-AgNPs) at different concentrations for 15 days in cutaneous wounds in rats experimentally infected with *C. albicans.* Control: *C. albicans* infected and non-treated rats, commercial cream: ketoconazole cream (2%), PEE-HA-AgNPsI: 25% concentration of the formulated nanocomposite, PPE-HA-AgNPsII: 50% concentration of the formulated nanocomposite, PPE-HA-AgNPsIII: full concentration of the formulated nanocomposite. ^a–e^Mean values with distinct letters in the same column changed significantly (*p* < 0.05).

The impact of PPE-HA-AgNPs at different concentrations on the molecular mechanisms involved in oxidative and antioxidant status is shown in **Figure 8**. The expression level of the *NQO1* gene was significantly increased (*p* < 0.05) in the PPE-HA-AgNPsIII-treated group compared with the control group. All the groups treated with PPE-HA-AgNPs at different concentrations showed higher expression levels (*p* < 0.05) of antioxidant-related genes (*CAT*, *SOD*, *GSH-Px*, *HO-1*, and *NQO1*) with simultaneous lower expression levels (*p* < 0.05) of the *Nrf2* gene. The relative mRNA expression of *CAT*, *GSH-Px*, *HO-1*, and *NQO1* reached their peaks (*p* < 0.05) in the group treated with PPE-HA-AgNPsIII (1.56, 1.67, 1.52, and 1.87-fold changes, respectively) in comparison with the control untreated group. In contrast, the groups treated with PPE-HA-AgNPsII and PPE-HA-AgNPsIII exhibited lower expression levels (*p* < 0.05) of the *Nrf2* gene (0.43 and 0.41- fold change, respectively) in comparison with control untreated group.

### 3.8 Investigating the healing and therapeutic impact of PPE-HA-AgNPs on angiogenic and wound healing markers

**Figure 9:**
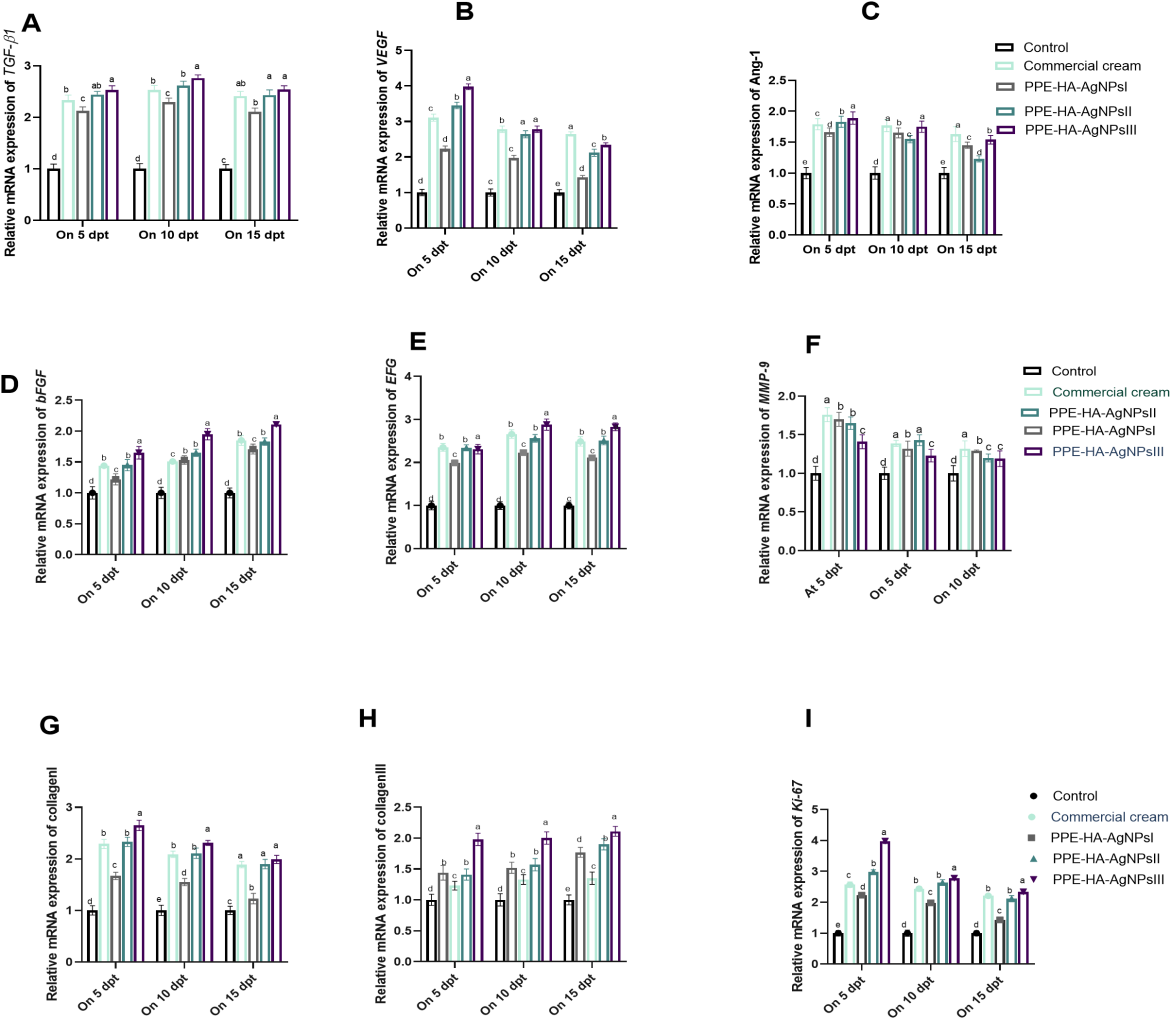
Relative mRNA expression of wound healing-related genes: *TGF-β1* [transforming growth factor beta 1, **(A)**], *VEGF* [vascular endothelial growth factor, **(B)**], *Ang-1* [angiopoietin-1, **(C)**], *bFGF* [basic fibroblast growth factor, **(D)**], EGF [epidermal growth factor, **(E)**], *MMP-9* [matrix metalloproteinase-9, **(F)**], collagen I **(G)**, collagen III **(H)**, and *Ki-*67 [Kiel-67, **(I)**] at three intervals [5, 10 and 15 days post-treatment (dpt)] in response to the therapeutic effects of a hydrogel loaded with pomegranate peel extract-hyaluronic acid-silver nanoparticles (PPE-HA-AgNPs) at different concentrations for 15 days in cutaneous wounds in rats experimentally infected with *C*. *albicans.* Control: *C. albicans* infected and non-treated rats, commercial cream: ketoconazole cream (2%), PEE-HA-AgNPsI: 25% concentration of the formulated nanocomposite, PPE-HA-AgNPsII: 50% concentration of the formulated nanocomposite, PPE-HA-AgNPsIII: full concentration of the formulated nanocomposite. ^a–e^Mean values with distinct letters in the same column changed significantly (*p* < 0.05).

On day 5, the *MMP-9* expression level was significantly (*p* < 0.05) decreased in all the topically treated groups compared with the control untreated group and it gradually decreased over time, especially in the PPE-HA-AgNPsII and PPE-HA-AgNPsIII-treated groups. Notably, the PPE-HA-AgNPsIII-treated group expressed higher levels of both collagen I and III on days 5 and 10 post-treatment. Meanwhile, on day 15 after-treatment, the groups treated with PPE-HA-AgNPsII, PPE-HA-AgNPsIII, and ketoconazole cream exhibited the same expression levels of the collagen I gene. Over time, the expression levels of the *VEGF* and *Ang-1* genes gradually decreased with an increase in the PPE-HA-AgNP dose, and the lowest value (*p* < 0.05) was recorded on day 15 post-treatment in the group topically treated with PPE-HA-AgNPsIII (**Figure 9**). Further RT-qPCR analysis revealed that the *TGF-β1*, *bFGF*, and *EGF* genes had markedly poor expression in the control untreated group. In comparison, their expression levels were increased, reaching their peaks in the PPE-HA-AgNPsIII-loaded-hydrogel-treated group on days 10 and 15 post-treatment. Moreover, the groups treated with PPE-HA-AgNPsIII and ketoconazole cream had no significant difference (*p* > 0.05) regarding the expression of the *TGF-β1* gene on day 15 post-treatment. Five days from the onset of treatment, the expression levels of the *Ki-*67 gene reached their maximum in the PPE-HA-AgNPsIII-treated group, followed by PPE-HA-AgNPsII and ketoconazole cream-treated groups. Furthermore, there was a dose-dependent reduction in *Ki-*67 gene expression with the lowest reduction found in the PPE-HA-AgNPsIII-treated rats on day 15 post-treatment (**Figure 9**).

**Figure 10:**
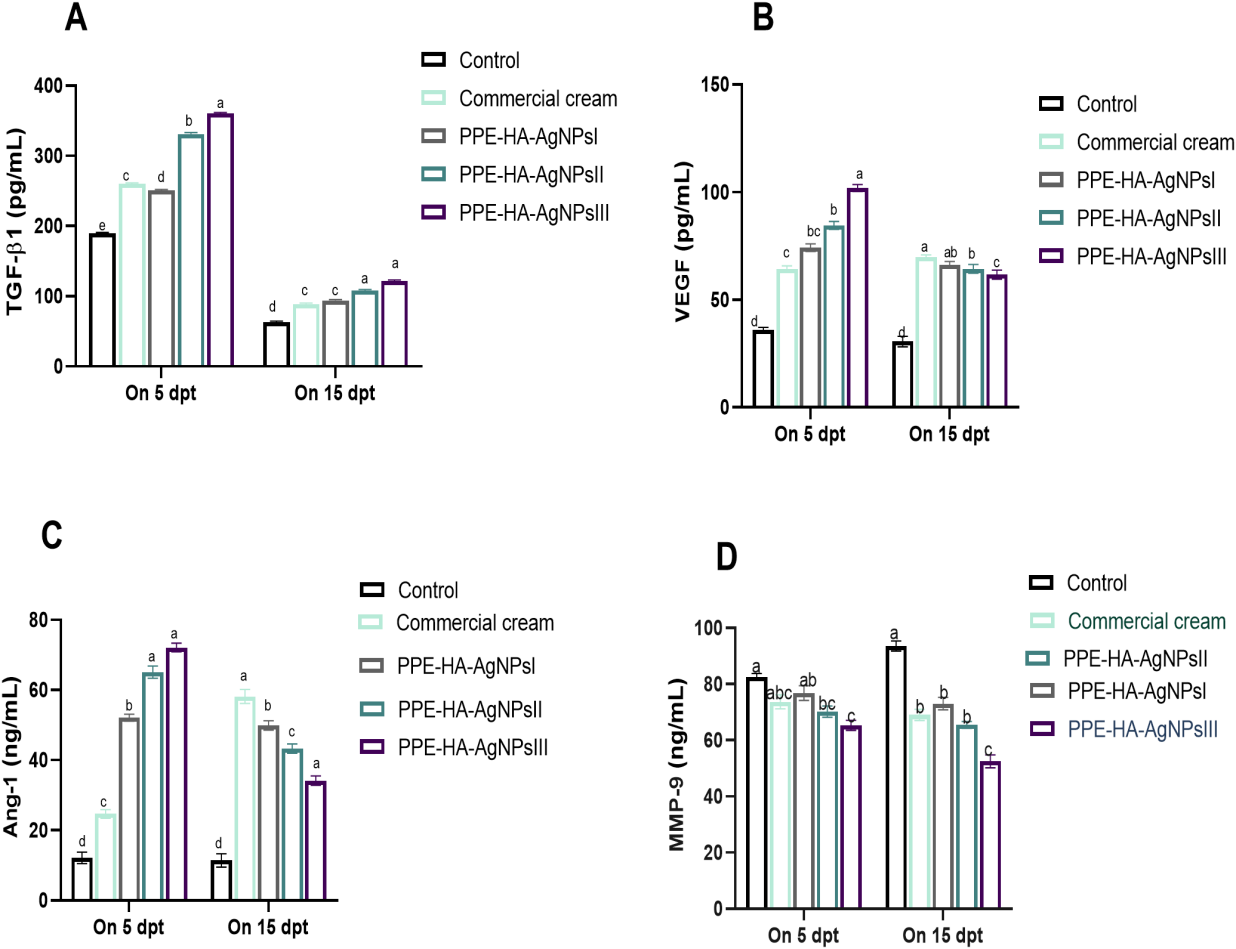
ELISA estimation of wound healing-related markers: TGF-β1 [transforming growth factor beta 1, **(A)**], VEGF [vascular endothelial growth factor, **(B)**], Ang-1 [angiopoietin-1, **(C)**], and MMP-9 [matrix metalloproteinase-9, **(D)**] at two intervals [5 and 15 days post-treatment (dpt)] in response to the therapeutic effects of hydrogel loaded with pomegranate peel extract-hyaluronic acid-silver nanoparticles (PPE-HA-AgNPs) at different concentrations for 15 days in cutaneous wounds in rats experimentally infected with *C*. *albicans.* Control: *C. albicans* infected and non-treated rats, commercial cream: ketoconazole cream (2%), PEE-HA-AgNPsI: 25% concentration of the formulated nanocomposite, PPE-HA-AgNPsII: 50% concentration of the formulated nanocomposite, PPE-HA-AgNPsIII: full concentration of the formulated nanocomposite. ^a–e^Mean values with distinct letters in the same column changed significantly (*p* < 0.05).

The effect of the PPE-HA-AgNP-loaded hydrogel on the angiogenic and wound healing markers, measured using ELISA assays, is shown in **Figure 10**. Five days after treatment, all groups were observed to have higher TGF-β1 levels than on day 15 post-treatment, where the biggest reduction was detected in the PPE-HA-AgNPsII and PPE-HA-AgNPsIII-treated groups compared to the control untreated group. All the treated groups had higher Ang-1 and VEGF levels on day 5 post-treatment and their levels were decreased on day 15 post-treatment, especially in the PPE- PPE-HA-AgNPsII-treated group. On day 5 post-treatment, all the PPE-HA-AgNP-treated groups exhibited lower *MMP-9* levels in a dose-dependent manner compared with the control untreated group with significant reduction in the PPE-HA-AgNPsIII-treated group on day 15 after treatment.

### 3.9 Histopathological examination

**Figure 11:**
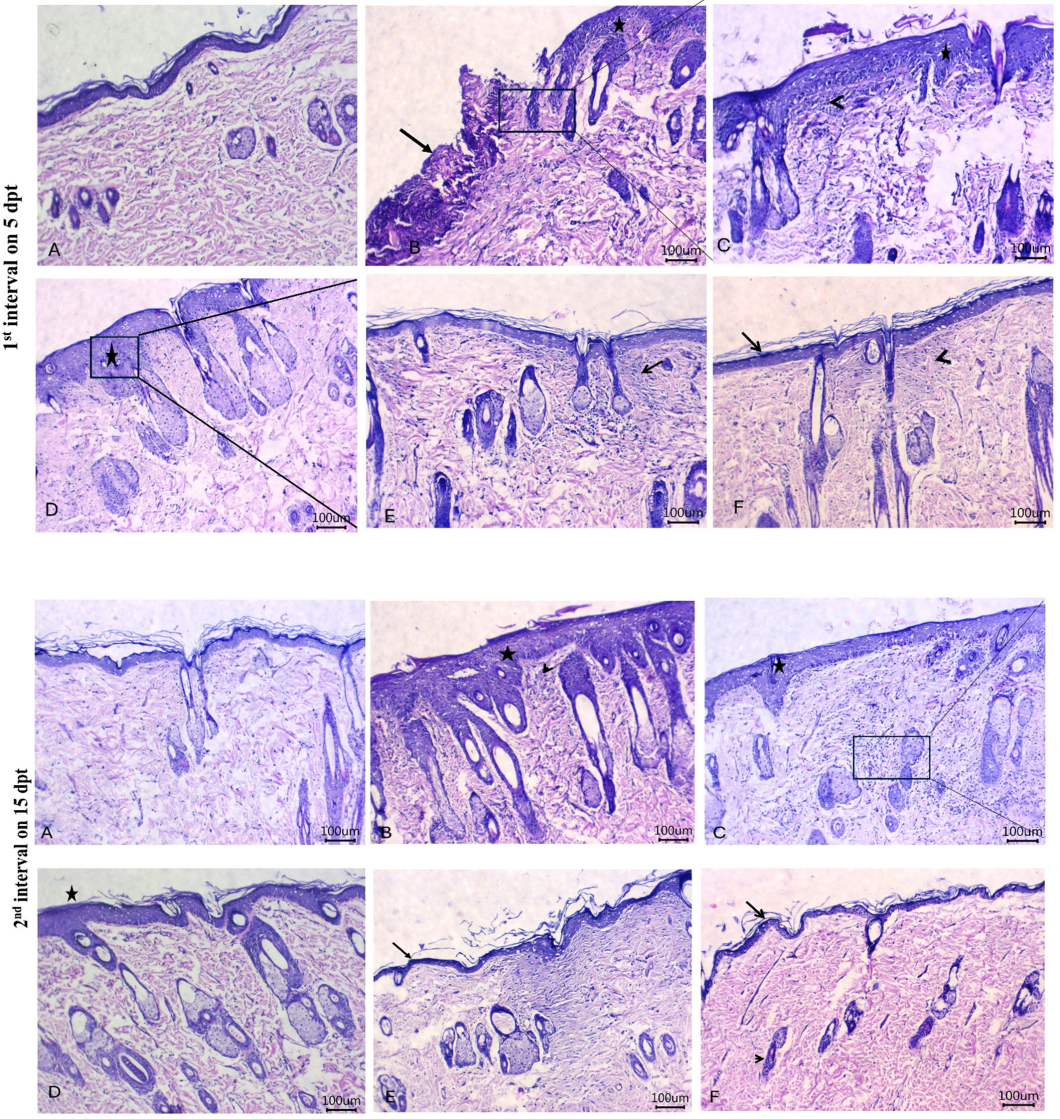
Histopathological images of skin tissues of rats experimentally infected with *C*. *albicans* in response to the therapeutic effects of a hydrogel loaded with pomegranate peel extract-hyaluronic acid-silver nanoparticles (PEE-HA-AgNPs) at different concentrations for 15 days on 5 (1^st^ interval) and 15 (2^nd^ interval) dpt (days post-treatment). 1^st^ interval: normal skin tissues of non-infected rats **(A)**; *C. albicans* infected and non-treated rats showing gap areas filled with crust or hyper-eosinophilic hyaline materials (arrow) with leukocytic infiltrates beside acanthosis (star) **(B)**; ketoconazole cream- (2%) treated rats showing acanthosis (star) with sub-epidermal inflammatory cells infiltrates (arrowhead) **(C)**; PEE-HA-AgNP- (25%) treated rats revealing intense acanthosis (star) with spongiosis (arrow) **(D)**; PEE-HA-AgNP- (50%) treated rats displaying apparent normal configurations of the epidermis, dermis, and skin appendages with few leukocytic infiltrates (arrow), and hyalinization (arrowhead) within the dermal layer **(E)**; and PEE-HA-AgNP- (100%) treated rats exhibiting normal structures of the epidermis (arrow), dermis, and skin appendages with a minute area of hyalinizing fibrous tissue within the superficial dermal layer (arrowhead) **(F)**. 2^nd^ interval: normal skin tissues of non-infected rats showing normal histomorphological structure of the epidermal layer with a superficial keratin layer and a normal dermal layer with associated skin appendages **(A)**; *C. albicans* infected rats showing marked acanthosis, spongiosis (star) with prominent finger projections toward the dermal layer beside dilated blood vessels and leukocytic infiltrates (arrowhead) within the dermal layer **(B)**; ketoconazole cream- (2%) treated rats showing moderate acanthosis (star) with minute round cells' infiltrates adjacent to sebaceous glands **(C)**; PPE-HA-AgNP- (25%) treated rats revealing mild acanthosis (star) and apparent normal architecture of skin appendages within the dermis **(D)**; PPE-HA-AgNP- (50%) treated rats displaying apparently normal epidermis (arrow) and dermis layers with hyalinizing or mature fibrous tissue within the superficial dermal layer (arrowhead) **(E)**; and PPE-HA-AgNP- (100%) treated rats exhibiting normal histological structures in the epidermis (arrow), dermis, and skin appendages (arrowhead) **(F)**. Scale bar 100 μm.

Histopathological images of the therapeutic effects of PEE-HA-AgNPs at different concentrations in the skin tissue of rats that were experimentally infected with *C. albicans* are shown in **Figure 11**. The control untreated group showed the most apparent changes in the skin tissue on day 5 following treatment in the form of a gap area filled with crust or hypereosinophilic hyaline materials that contained filamentous elements, reflecting the presence of *C. albicans*, mixed with leukocytic infiltrates when compared with normal skin histomorphological structure in **Figure 11A**. Moreover, there was acanthosis, distorted skin appendages, and dermatitis adjacent to this gap area. Meanwhile, on day 15 post-treatment, there was marked acanthosis, spongiosis with prominent finger projections toward the dermal layer beside dilated blood vessels, and leukocytic infiltrates within the dermal layer (**Figure 11B**). Treatment with the ketoconazole cream showed prominent acanthosis with sub-epidermal inflammatory cell infiltrates and dermal edema on day 5 post-treatment and moderate acanthosis with minute round cell infiltrates adjacent to the sebaceous glands on day 15 post-treatment (**Figure 11C**). Following treatment with PPE-HA-AgNPsI, intense acanthosis with spongiosis and minute dermal leukocytic infiltrates were encountered on day 5 after-treatment, and mild acanthosis and apparent normal architecture of skin appendages within the dermis were found on day 15 post-treatment (**Figure 11D**). Treatment with PPE-HA-AgNPsII resulted in apparent normal configurations of the epidermis, dermis, and skin appendages with few leukocytic infiltrates and hyalinization within the dermal layer on day 5 following treatment and an improvement in the integrity of the skin layers with hyalinizing or mature fibrous tissue within the superficial dermal layer on day 15 post-treatment (**Figure 11E**). Treatment with PPE-HA-AgNPs at the full concentration relieved the histopathological lesions in *C. albicans*-infected rats evidenced by the normal structure of the epidermis, dermis, and skin appendages with a minute area of hyalinizing fibrous tissue within the superficial dermal layer on day 5 after treatment and histologically normal structures of the epidermis, dermis, and skin appendages on day 15 post-treatment (**Figure 11F**).

## 4 Discussion

Wound healing problems are serious therapeutic challenges among other healthcare concerns. Proper cutaneous wound healing is fundamental for the restoration of disrupted anatomical stability and the functional status of the skin (Okur et al., 2020). However, improper wound healing could result in the invasion of pathogenic microorganisms with progression to disadvantageous clinical complications and increased risk to the patients and treatment costs (Scappaticci et al., 2021). *C. albicans* is the most prevalent human pathogen worldwide and a major contributor to wound infections (Brown et al., 2012), and it is considered an emerging resistant fungal pathogen of the skin that can result in life-threatening infections with possible mortality in healthcare settings and hospitals (Gil et al., 2022). Up till now, antifungal therapy has been restricted for *C. albicans* due to the emergence of drug resistance against the current antifungals and the low effectiveness of topical drug penetration. Therefore, the development of novel efficient evidence-based therapy, combining nanotechnology and phytotherapy, for candidiasis, and in particular, those with long-lasting effects and targeting biofilms is urgently required. Thus, the current study showed that nanocomposite-loaded hydrogel therapy combining PPE, HA, and AgNPs can effectively exert antifungal activity against *C. albicans*, and it was found to be efficient in wound healing. One important factor that contributes to the pathogenesis of candidiasis is biofilm formation as *C. albicans* has the ability to form biofilms on both inert and biological surfaces (Taff et al., 2013). The current study showed that the infected and non-treated group exhibited the greatest colonization and invasion of *C. albicans* inside or around the wound area, while the lowest colonization and invasion were detected in the group treated with the highest concentration of PPE-HA-AgNPs. Additionally, the *C. albicans* biofilm was inhibited by a 55% concentration of PPE, which is mainly attributed to its phenolic compounds (de Almeida Rochelle et al., 2016). The aforementioned findings reveal that the combination of HA-AgNPs and PPE in a nano hydrogel composite exhibited effective fungicidal activity.

In comparison with the group treated with a commercial cream of azole derivatives, the group treated with PPE-HA-AgNPs at full concentration showed higher antifungal and antibiofilm potency, which were evidenced by a lower *C. albicans* burden and the downregulation of its biofilm-related genes. Accordingly, the anti-biofilm effects of AgNPs treatment were outer cell membrane disruption and *C. albicans* filament inhibition (Lara et al., 2015). The mechanistic action of metallic NPs involves the disruption of the cell walls, leading to an increase in their permeation owing to the electrostatic interaction between positively charged NPs and negatively charged cell wall molecules of the microorganism, resulting in cytoplasmic content leakage (Joshi et al., 2020) and membrane potential disorders (Taff et al., 2013). Additionally, AgNPs exert their anti-fungal activity via the permeabilization of target cell membranes, destruction of proton pumps, and denaturation of proteins after the interaction with phosphoric and sulfur groups that exist on the surface of the fungal cell wall (Dulińska-Litewka et al., 2021). Furthermore, treatment with erodium glaucophyllum loaded-AgNPs resulted in declines in the counts of fungal cells and lesion rates, suggesting their prospective use in the treatment of oral candidiasis (Abdallah and Ali, 2022). Additionally, metallic NPs can potentiate ROS generation, as another mechanism for killing pathogenic microorganisms (Canaparo et al., 2020) which aligns with our findings following the treatment with PPE-HA-AgNPs. The fungicidal capacity of AgNPs was explained by their aggregation outside the fungal cells, the release of their silver ions, and thus the generation of cell death via the reduction process resulting from the interaction of ionic silver with cell components (Vazquez-Muñoz et al., 2014). Together with the antifungal efficacy of AgNPs, recent studies have revealed that PPE is an attractive natural anti-fungal alternative against wide varieties of fungal pathogens owing to its active inhibitors including phenolics and flavonoids (Dahham et al., 2010; Sadeghian et al., 2011). Additionally, the significant inhibitory antifungal effect of pomegranate water extract against *C. albicans* has been reported (Tayel and El-Tras, 2010). The anticandidal potentiality of a free form of PPE in a previous study was explained by the synergistic effect of their combined constituents, which include powerful antibacterial and antifungal compounds (Seeram et al., 2006). In the current study, the long-lasting antifungal impact and optimal functionality of the novel nanocomposite formulated with PPE-HA-AgNPs are linked to its higher biodegradability and the controlled release of active biomolecules in the infected wound area, which accelerate healing.

The wound healing process involves multiple distinct and overlapping inflammatory and granulation phases, including hemostasis/inflammation, proliferation, fibrogenesis, neovascularization, wound contraction, and epithelialization (Wang et al., 2018). During the course of an injury, the inflammation process assists by eliminating damaged cells and promoting vasoconstriction (Yuniarti et al., 2018). Furthermore, the recruitment of immune cells such as neutrophils and macrophages results in the production of proinflammatory mediators such as interleukins and cytokines and the equilibrated scenario of inflammatory response generally happens. However, prolonged inflammatory events can become a chronic situation that is dangerous for the wound-healing process (Kiritsi and Nyström, 2018). In the current study, the combined curative impact of PEE, HA, and AgNPs not only reduced fungal colonization in the skin tissue of the rat model but also decreased the excessive inflammatory response associated with infected wounds. Herein, the groups topically treated with higher concentrations of PPE-HA-AgNPs showed decreased exaggerated inflammatory responses that triggered tissue damage during wound healing as proved by the reduction in the levels of *TNF-α*, *IL-6*, and *IL-1β* in the skin in a dose-dependent manner, especially at 10 days post-treatment, which was in conjunction with the downregulation of cytokine-related genes (*IL-1β*, *IL-6*, *IL-18*, and *TNF-α*). In addition, the immune-stimulatory role of PPE-HA-AgNPs was shown by the increase in anti-inflammatory cytokine production, especially IL-10. TNF-α, IL-6, and IL-1β are proinflammatory cytokines that are over-secreted and expressed during the inflammatory phase in a variety of disease states (Scheller et al., 2011). The inhibition of aggravated expression of proinflammatory cytokines and boosting the expression of anti-inflammatory cytokines are essential healing mechanisms (Abraham and Kappas, 2008). In the same way, using natural compounds as a substitute for chemical ones triggers healing with satisfactory results (Dwivedi et al., 2017; Shen et al., 2017).The use of pomegranate extracts to accelerate the healing process has previously been described (Elzayat et al., 2018; Lukiswanto et al., 2019).

Moreover, the antimicrobial and anti-inflammatory properties of pomegranate are due to the presence of phenolic acids and flavonoids that have an active role in wound healing (Salama et al., 2021). Moreover, HA possesses the capability to inhibit inflammatory events such as cytokine and prostaglandin production (Shalumon et al., 2018). The anti-inflammatory properties of pomegranate extracts are of great interest, and punicalagin is highly implicated in this anti-inflammatory ability (El-Missiry et al., 2015). Moreover, pomegranate extract with 40% ellagic acid accelerated the healing of deep second-degree burn wounds due to its anti-inflammatory properties (Lukiswanto et al., 2019). The anti-inflammatory properties of acetone extract from whole pomegranate fruit resulted in phosphorylation inhibition of the release of numerous cytokines, and the mechanism underlying this was observed to be NF-κB-dependent (Colombo et al., 2013). Furthermore, punicalagin in PPE can regulate signaling pathways in inflammation-associated disorders by reducing the production of TNFα-induced expressions of pro-inflammatory cytokines, including IL-1β, IL-6, and IL-8 (BenSaad et al., 2017; Venusova et al., 2021). In addition, AgNPs could reduce inflammation by inhibiting the release of pro-inflammatory cytokines, such as IL-6, and decreasing CRP levels, thereby promoting wound repair (Liu et al., 2010). Another beneficial outcome, beyond the topical application of PPE-HA-AgNPs, is the regulation of redox balance in infected wounds through the modulation of ROS and antioxidant levels. The inflammatory process in infected wounds can induce oxidative stress and reduce cellular antioxidant capacity and, in this way, exaggerated ROS production can distribute redox homeostasis membrane lipids and modulate DNA and protein structures (Dwivedi et al., 2014). In addition, H_2_O_2_ is a prominent secondary intracellular messenger that regulates various stages of wound healing including cell recruitment, cytokine production, cell proliferation and migration, and angiogenesis (Bryan et al., 2012). Moreover, HO-1 is an enzyme that dissociates heme and produces antioxidant molecules, iron ions, and carbon monoxide that control the principal processes involved in apoptosis, inflammation, and cell angiogenesis and proliferation (Loboda et al., 2016). Throughout wound healing, Nrf2 decreases oxidative stress in cells, has a fundamental role in the proliferation of epithelial cells’ apoptosis and migration (Long et al., 2016), and controls MMP-9 levels (Jindam et al., 2017). Interestingly, in the present study, we proved that PPE-HA-AgNPs topical application enhanced antioxidant status and notably decreased oxidative stress biomarkers compared with the control untreated or even commercially treated groups. These findings were supported by a significant increase in SOD, GSH-Px, and catalase activity and TAC levels with a simultaneous decrease in ROS, H_2_O_2_, and MDA levels in the skin tissues. At the molecular level, a significant upregulation of HO-1, NQO1, Nrf2, and other antioxidant-related genes was detected following the administration of PPE-HA-AgNPs, which aligns with the findings of Sayed et al. (2022). Similarly, the antioxidant functions of the active constituents in pomegranate can counteract oxidative stress by maintaining MDA, glutathione, catalase, and peroxidase levels (Kaur et al., 2015). Sreekumar et al. (2014) stated that combining pomegranate with silver promotes wound healing, accelerates epithelialization, and enhances remodeling due to the free radical scavenging and antioxidant activity of polyphenols. Additionally, the quantification of wound fluid nitrate in the form of NO bioactivity in the wound healing process suggested a diagnostic role in good wound healing (Boykin, 2010). Emerging evidence indicates that NO plays a central role in wound healing, including angiogenesis and migration and proliferation of fibroblasts, epithelial cells, endothelial cells, and keratinocytes (Gallagher et al., 2007). It also facilitates intercellular communication to regulate cell proliferation and collagen production (Witte and Barbul, 2002). An optimal release of NO would improve wound healing; however, over-production of NO over time can result in the development of chronic wounds (Barrientos et al., 2008). In accordance with these findings, the NO and NOS activity in PPE-HA-AgNP-treated rats was lower than that in the non-treated ones, especially at 10 days after treatment. These potentiated antioxidant outcomes of PPE-HA-AgNPs are mainly owing to the polyphenolic molecules of the pomegranate peel extract which exert potent antioxidant activities with wound healing and antiallergic properties that reduce skin damage via their free radical scavenging power, which is in accordance with Salama et al. (2021). Additionally, the nanoformulation of PPE can prolong the release of active substances, thus optimizing its therapeutic delivery (Paczkowska-Walendowska et al., 2024). On the other hand, the remarkable effect of PPE-HA-AgNPs was explained by the gelling characteristics of HA, which acts as a hydrophobic agent that augments the efficacy of AgNPs due to the enhancement of the transportation mechanism via the cellular membrane with prolonged therapeutic activity owing to its size (Ivashchenko et al., 2018). Moreover, the prolonged pharmacological effect in rats of HA is a result of the drug release and delivery in a nano-form (Liu et al., 2018). Besides, Makvandi and co-workers (Makvandi et al., 2019) showed that hydrogels comprising AgNPs, HA, and corn silk extract exhibited adequate biocompatibility. Additionally, their application brings about excellent wound closure and a smaller wound area compared to the control samples in a wound healing assay. Another explanation is that wound dressings with a combination of nano-silver and HA are extremely hygroscopic, facilitating the removal of excessive exudates from the wound beds (El-Aassar et al., 2020). They also have an excellent rate of water-vapor transmission and are consequently capable of preventing dryness and dehydration of wounds, providing an appropriate environment for the regeneration of skin tissues (Tarusha et al., 2018). Moreover, the effectiveness of PPE-HA-AgNPs at a higher concentration was supported by histopathological examination that showed a decrease in exaggerated inflammatory response, replacement of necrotic tissues with granulation ones along with reduced cellularity, and increased collagen deposition and skin re-epithelialization. In accordance, a methanolic extract of pomegranate peels had an enhanced healing capability by ameliorating the histopathological picture in excision cutaneous wounds in diabetic rats (Karim et al., 2021). Additionally, pomegranate fractions promote proliferation, procollagen synthesis, and thus the regeneration of the dermis and epidermis (Aslam et al., 2006). Moreover, another study that utilized AgNPs and pomegranate formulations in diabetic rats showed that the quantification of inflammatory infiltrates was greatly reduced in histological pictures after treatment (Scappaticci et al., 2021).

During tissue repair, inflammatory cells stimulate the migration and proliferation of endothelial cells, leading to neovascularization. This process involves connective tissue cells synthesizing extracellular matrices, including collagen, as well as keratinocytes, which contribute to the re-epithelialization of the wounded tissue (Olczyk et al., 2014). Collagen, a major extracellular protein matrix, is the component that ultimately contributes to wound strength (Chithra et al., 1998). Additionally, collagen molecules play a fundamental role in wound contraction and maintaining tissue matrix integrity, both of which are crucial for effective wound healing. Types I and III collagen are the primary types involved in wound repair, and their ratio remains constant in normal skin. However, in mature scar tissue formed after trauma, the ratio of type I collagen is greater than that in normal skin (Hu et al., 2023). In line with these findings, the rats in the groups topically treated with PPE-HA-AgNP-loaded hydrogel showed enhanced healing signs for cutaneous wounds via improvement in wound size, contraction, and closure together with a lower expression of the *MMP* gene and higher expression of the collagen I and III genes. This revealed increased fibroblast proliferation and collagen regeneration that provided negative entities for pathogenic microorganisms such as *C. albicans*, promoting wound healing. In accordance, a free form of a water-soluble extract made from pomegranate peel was found to inhibit the elaboration of the major MMP-1 enzyme which is responsible for collagen destruction in aged/photoaged skin (Brennan et al., 2003). Moreover, it was found that MMP-1 accumulation in the fibroblast-conditioned medium was dramatically reduced in the presence of the pomegranate peel extract (Aslam et al., 2006). Additionally, a therapeutic synergistic strategy based on Au/Ag nanodots resembling a pomegranate-like core in a polyvinyl alcohol hydrogel had an efficient treatment impact on diabetic wounds via their superior anti-inflammatory properties with accelerated collagen deposition capacity (Wang et al., 2023).

Additionally, an imbalance of cytokine expression and growth factors, increased activity of metalloproteinase, and oxidative stress can impair neo-angiogenesis and result in cell dysfunction and wound healing retardation (Bannon et al., 2013). During the process of natural wound healing, angiogenic activity primarily results in the formation of a disorganized vascular network at the injury site, characterized by an increased number of blood vessels. Once vessel density reaches its peak, vascular remodeling occurs, marked by the regression of the vascular network and the gradual maturation of blood vessels (Urciuolo et al., 2019). Many angiogenic growth factors and signaling pathways are involved in early vascular development, among these are VEGF, bFGF, Ang, and TGF-β (Crivellato, 2011). VEGF appears to be a key factor in pathological events such as tissue repair, which involves neovascularization and increased vascular permeability (Santos et al., 2007). Moreover, it improves angiogenesis during wound healing by stimulating the migration of endothelial cells through the extracellular matrix. In contrast, abnormal angiogenesis can result from inflammatory stimulation (Hu et al., 2023). FGF signaling has a critical role in efficient healing, while its deficiency reflects a state of wound healing impairment (Ortega et al., 1998). Furthermore, TGF-β can promote cells to enhance extracellular matrix protein synthesis and concurrently reduce collagen proteases (El Gazaerly et al., 2013). Herein, significant increases in pro-angiogenic markers reflected by the higher expression of the *Ang-1* and *VEGF* genes in the created cutaneous wounds treated with PPE-HA-AgNPs were remarkably detected between 5 dpt and 10 dpt and gradually decreased by 15 dpt. Moreover, a significant increase in re-epithelialization markers together with deposition of collagen fibers, which were supported by the upregulation of *TGF-β1*, *bFGF*, *EFG*, and collagen I and III genes post-treatment with a higher concentration of PPE-HA-AgNPs, indicated faster and successful wound healing. These findings were also supported by the ELISA results for markers associated with wound healing as established in an earlier study (Zhou et al., 2017). We hypothesized that the presence of PPE-HA-AgNPs enhanced TGF-β availability in the extravascular environment by priming cells, boosting their response to the normal regulatory factors at the site of injury. These findings were in agreement with a previous study (Özkan et al., 2004), where in the first week after injury, higher neovascularization was crucial for providing oxygen and necessary nutrients to the wound site and initiated granulation tissue formation.

In the same context, Elbialy et al. (2020) found that the significant gene expression levels of *bFGF* and *VEGF* to the remarkable wound healing process led to enhanced angiogenesis and collagen deposition with improved epithelialization rate. These results can be linked to the scavenging effects of PPE-enriched flavonoids, as proven by the low ROS levels detected in the current study, which are required to activate cell signaling pathways and angiogenesis, participating in the elimination of invading pathogens. Inversely, high ROS levels in the non-treated rats triggered oxidative stress that seriously compromised tissue repair, resulting in unhealed wounds that can be painful and require expensive and long-term treatments as proven previously (Dunnill et al., 2017). Similarly, given the potent angiogenic and antioxidant activities of flavonoids, unique sets of nanocomposite membranes delivering them were prepared for wound-healing applications (Lee et al., 2014). Proper wound healing was also reinforced by HA naturally occurring in the extracellular matrix with structural and biological properties mediating its activity in the matrix organization (Um et al., 2004). In accordance, epigallocatechin gallate enhanced diabetic wound healing by activating the expression of angiogenesis-related genes and decreasing the expression of inflammation-related genes (Hu et al., 2023). A herbal extract enriched with ellagic acid is expected to accelerate angiogenesis-related markers (Nirwana et al., 2022). In addition, angiogenic activity by an active ingredient of PPE such as punicalagin in this study could contribute to the processes of tissue repair and wound healing as previously documented by Carneiro et al. (2016). Furthermore, ELISA assays supported by qRT-PCR results revealed that VEGF expression in wound tissues of rats treated with a methanolic extract based-gel from a Saudi pomegranate peel gel was significantly higher than that in diabetic non-treated rats (Karim et al., 2021). Moreover, Dai et al. (2021) stated that matrix metalloproteinase plays an important role in the normal wound remodeling process as it can enzymatically dissociate tissue extracellular matrix and mediate cell migration, while excessive MMP-9 production can prevent wound healing. Herein, the expression of the *MMP-9* gene in wound tissue specimens of rats following treatment with PPE-HA-AgNPs was dose-dependently lower than that in the traditionally treated rats. In accordance, various bioactive pomegranate fractions might inhibit the elaboration of the major enzyme, MMP, which is responsible for skin collagen destruction, in agreement with previous studies (Aslam et al., 2006; Brennan et al., 2003). Increased cell proliferation is a crucial aspect of wound healing, in general, and the Ki-67 protein is an important marker of this cellular event. Ki-67 expression is also widely known to be an indicator of cell growth within a total cell population (Choi et al., 2012). The current study established an increased expression of *Ki-67* gene with an increasing dose of PPE-HA-AgNPs compared with the traditionally treated group suggesting that the prepared nanocomposite increased cell proliferation and the consequent stimulus for the remodeling phase. Furthermore, the beneficial effects of the formulated nanocomposite could result from its incorporation into an efficient nanocarrier that stimulates the abovementioned cellular and molecular processes, aiding in the wound microenvironment. This was owing to its distinct antimicrobial, anti-inflammatory, and angiogenic effects and better adsorption and loading capacity that possibly changed the wound’s milieu from a non-healing to a healing state (Kushwaha et al., 2022).

The aforementioned findings were also supported by the healing effect of the PPE-HA-AgNPs on wound tissue regeneration in the histological examinations that demonstrated an improvement in the integrity of the skin layers with hyalinizing or mature fibrous tissue within the superficial dermal layer on day 15 post-treatment. In contrast, the untreated infected group showed the most pronounced changes in the skin tissues of rats on day 5 post-treatment, characterized by gap areas filled with crusts or hyper-eosinophilic hyaline materials containing filamentous elements, indicative of the presence of *C. albicans*. Furthermore, marked acanthosis, spongiosis with prominent finger projections toward the dermal layer beside dilated blood vessels, leukocytic infiltrates within the dermal layer, and distorted skin appendages were detected in this group. Notably, the higher concentration of PPE-HA-AgNPs caused a complete regeneration of the epidermis and dermis, and a new epithelium was visible as apparent skin appendages with prominent wound contraction and no evidence of filamentous elements reflecting the presence of *C. albicans*. Similarly, post-AgNDs@Gel + NIR treatment, the histological view of skin wounds was improved with complete healing, evidenced by enhanced formation of epidermis and dermis tissue (Wang et al., 2023).

## 5 Conclusion

The favorable effects of PPE-HA-AgNPs on the healing of *C. albicans* infected wounds seem to be mainly due to the innovative nano-delivery system. PPE exerts its potential functions by reducing the ability of free radicals to generate tissue damage, augmenting antioxidant status, and diminishing inflammatory markers with enhanced collagen deposition and antifungal activity. Moreover, the mechanism of action of metallic AgNPs against *C. albicans* was found to be the modification of biofilm-specific genes which maintain sustained inflammation and delay wound healing. Based on our results, we conclude that PPE-HA-AgNPs accelerate the healing of cutaneous wounds infected with *C. albicans* via various mechanisms including an extended-release with better retention ability on the skin surface, reducing the exaggerated inflammatory response, and promoting new granulation tissues, angiogenesis, and skin re-epithelialization. Therefore, our findings demonstrate the wound-healing potential of PPE-HA-AgNPs as a promising therapeutic agent against cutaneous wounds infected with *C. albicans*.

## Data Availability

The original data presented in the study are included in the article/supplementary material. Further inquiries can be directed to the corresponding author.

## References

[B1] Abdallah B. M. Ali E. M. (2022). Therapeutic Effect of Green Synthesized Silver Nanoparticles Using Erodium glaucophyllum Extract against Oral Candidiasis: *In Vitro* and *In Vivo* Study. Molecules 27, 4221. doi: 10.3390/molecules27134221 35807474 PMC9267989

[B2] Abou Hammad A. B. Hemdan B. A. El Nahrawy A. M. (2020). Facile synthesis and potential application of Ni0. 6Zn0. 4Fe2O4 and Ni0. 6Zn0. 2Ce0. 2Fe2O4 magnetic nanocubes as a new strategy in sewage treatment. J. Environ. Manage 270, 110816. doi: 10.1016/j.jenvman.2020.110816 32501235

[B3] Abraham N. G. Kappas A. (2008). Pharmacological and clinical aspects of heme oxygenase. Pharmacol. Rev. 60, 79–127. doi: 10.1124/pr.107.07104 18323402

[B4] Aggarwal B. B. Gupta S. C. Sung B. (2013). Curcumin: an orally bioavailable blocker of TNF and other pro-inflammatory biomarkers. Br. J. Pharmacol. 169, 1672–1692. doi: 10.1111/bph.2013.169.issue-8 23425071 PMC3753829

[B5] Aldakheel F. M. Sayed M. M. E. Mohsen D. Fagir M. H. El Dein D. K. (2023). Green synthesis of silver nanoparticles loaded hydrogel for wound healing; systematic review. Gels 9, 530. doi: 10.3390/gels9070530 37504410 PMC10378855

[B6] Alherz F. A. Negm W. A. Elekhnawy E. El-Masry T. A. Haggag E. M. Alqahtani M. J.. (2022). Silver nanoparticles prepared using encephalartos laurentianus de wild leaf extract have inhibitory activity against candida albicans clinical isolates. J. Fungi 8, 1005. doi: 10.3390/jof8101005 PMC9604723 36294570

[B7] Aslam M. N. Lansky E. P. Varani J. (2006). Pomegranate as a cosmeceutical source: Pomegranate fractions promote proliferation and procollagen synthesis and inhibit matrix metalloproteinase-1 production in human skin cells. J. Ethnopharmacol 103, 311–318. doi: 10.1016/j.jep.2005.07.027 16221534

[B8] Bancroft J. D. Gamble M. (2008). Theory and Practice of Histological Techniques. 6th Edition, Churchill Livingstone, London: Elsevier.

[B9] Bannon P. Wood S. Restivo T. Campbell L. Hardman M. J. Mace K. A. (2013). Diabetes induces stable intrinsic changes to myeloid cells that contribute to chronic inflammation during wound healing in mice. Dis. Model. Mech. 6, 1434–1447. doi: 10.1242/dmm.012237 24057002 PMC3820266

[B10] Barrientos S. Stojadinovic O. Golinko M. S. Brem H. Tomic-Canic M. (2008). Growth factors and cytokines in wound healing. Wound Repair Regener. 16, 585–601. doi: 10.1111/j.1524-475X.2008.00410.x 19128254

[B11] Barros M. Santos D. Hamdan J. S. (2007). Evaluation of susceptibility of Trichophyton mentagrophytes and Trichophyton rubrum clinical isolates to antifungal drugs using a modified CLSI microdilution method (M38-A). J. Med. Microbiol. 56, 514–518. doi: 10.1099/jmm.0.46542-0 17374893

[B12] BenSaad L. A. Kim K. H. Quah C. C. Kim W. R. Shahimi M. (2017). Anti-inflammatory potential of ellagic acid, gallic acid and punicalagin A&B isolated from Punica granatum. BMC Complement Altern. Med. 17, 1–10. doi: 10.1186/s12906-017-1555-0 28088220 PMC5237561

[B13] Boykin J. V. Jr (2010). Wound nitric oxide bioactivity: a promising diagnostic indicator for diabetic foot ulcer management. J. Wound Ostomy Continence Nurs. 37, 25–32. doi: 10.1097/WON.0b013e3181c68b61 20075688

[B14] Brennan M. Bhatti H. Nerusu K. C. Bhagavathula N. Kang S. Fisher G. J.. (2003). Matrix metalloproteinase-1 is the major collagenolytic enzyme responsible for collagen damage in UV-irradiated human skin. Photochem. Photobiol. 78, 43–48. doi: 10.1562/0031-8655(2003)078<0043:MMITMC>2.0.CO;2 12929747

[B15] Brown G. D. Denning D. W. Gow N. A. Levitz S. M. Netea M. G. White T. C. (2012). Hidden killers: human fungal infections. Sci. Transl. Med. 4, 165rv113–165rv113. doi: 10.1126/science.1222236 23253612

[B16] Brown P. K. Qureshi A. T. Moll A. N. Hayes D. J. Monroe W. T. (2013). Single-cell analysis using hyperspectral imaging modalities. ACS nano. 7, 2948. doi: 10.1021/nn304868y 23473419

[B17] Bruna T. Maldonado-Bravo F. Jara P. Caro N. (2021). Silver nanoparticles and their antibacterial applications. Int. J. Mol. Sci. 22, 7202. doi: 10.3390/ijms22137202 34281254 PMC8268496

[B18] Bryan N. Ahswin H. Smart N. Bayon Y. Wohlert S. Hunt J. A. (2012). Reactive oxygen species (ROS)–a family of fate deciding molecules pivotal in constructive inflammation and wound healing. Eur. Cell Mater 24, e65. doi: 10.22203/eCM.v024a18 23007910

[B19] Canaparo R. Foglietta F. Limongi T. Serpe L. (2020). Biomedical applications of reactive oxygen species generation by metal nanoparticles. Materials 14, 53. doi: 10.3390/ma14010053 33374476 PMC7795539

[B20] Carneiro C. C. da Costa Santos S. de Souza Lino R. Jr Bara M. T. F. Chaibub B. A. de Melo Reis P. R.. (2016). Chemopreventive effect and angiogenic activity of punicalagin isolated from leaves of Lafoensia pacari A. St.-Hil. Toxicol. Appl. Pharmacol. 310, 1–8. doi: 10.1016/j.taap.2016.08.015 27546523

[B21] Cheng G. Wozniak K. Wallig M. A. Fidel P. L. Jr. Trupin S. R. Hoyer L. L. (2005). Comparison between Candida albicans agglutinin-like sequence gene expression patterns in human clinical specimens and models of vaginal candidiasis. Infect. Immun. 73, 1656–1663. doi: 10.1128/IAI.73.3.1656-1663.2005 15731066 PMC1064955

[B22] Chithra P. Sajithlal G. Chandrakasan G. (1998). Influence of Aloe vera on collagen characteristics in healing dermal wounds in rats. Mol. Cell Biochem. 181, 71–76. doi: 10.1023/A:1006813510959 9562243

[B23] Choi D. S. Kim S. Lim Y.-M. Gwon H.-J. Park J. S. Nho Y.-C.. (2012). Hydrogel incorporated with chestnut honey accelerates wound healing and promotes early HO-1 protein expression in diabetic (db/db) mice. Tissue Eng. Regenerative Med. 9, 36–42. doi: 10.1007/s13770-012-0036-2

[B24] Clinical and Laboratory Standards Institute (2012). Methods for Dilution Antimicrobial Susceptibility Tests for Bacteria That Grow Aerobically; Approved Standard—Ninth Edition. CLSI document M07-A9. Pennsylvania, United States: Clinical and Laboratory Standards Institute.

[B25] Colombo E. Sangiovanni E. Dell′ Agli M. (2013). A review on the anti-inflammatory activity of pomegranate in the gastrointestinal tract. Evidence-Based Complementary Altern. Med. 2013, 247145. doi: 10.1155/2013/247145 PMC3612487 23573120

[B26] Crivellato E. (2011). The role of angiogenic growth factors in organogenesis. Int. J. Dev. Biol. 55, 365–375. doi: 10.1387/ijdb.103214ec 21858761

[B27] Dahham S. S. Ali M. N. Tabassum H. Khan M. (2010). Studies on antibacterial and antifungal activity of pomegranate (Punica granatum L.). Am. Eurasian J. Agric. Environ. Sci. 9, 273–281.

[B28] Dai J. Shen J. Chai Y. Chen H. (2021). IL-1β impaired diabetic wound healing by regulating MMP-2 and MMP-9 through the p38 pathway. Mediators Inflammation 2021, 1–10. doi: 10.1155/2021/6645766 PMC8149221 34054346

[B29] Das A. Kumar A. Patil N. B. Viswanathan C. Ghosh D. (2015). Preparation and characterization of silver nanoparticle loaded amorphous hydrogel of carboxymethylcellulose for infected wounds. Carbohydr Polym. 130, 254–261. doi: 10.1016/j.carbpol.2015.03.082 26076624

[B30] de Almeida Rochelle S. L. Sardi J. Freires I. A. de Carvalho Galvão L. C. Lazarini J. G. de Alencar S. M.. (2016). The anti-biofilm potential of commonly discarded agro-industrial residues against opportunistic pathogens. Ind. Crops Products 87, 150–160. doi: 10.1016/j.indcrop.2016.03.044

[B31] de Oliveira J. R. de Castro V. C. Vilela P. D. G. F. Camargo S. E. A. Carvalho C. A. T. Jorge A. O. C.. (2013). Cytotoxicity of Brazilian plant extracts against oral microorganisms of interest to dentistry. BMC Complement. Altern. Med. 13, 1–7. 23945270 10.1186/1472-6882-13-208 PMC3751599

[B32] Dulińska-Litewka J. Dykas K. Felkle D. Karnas K. Khachatryan G. Karewicz A. (2021). Hyaluronic acid-silver nanocomposites and their biomedical applications: A review. Materials 15, 234. doi: 10.3390/ma150102342 35009380 PMC8745796

[B33] Dunnill C. Patton T. Brennan J. Barrett J. Dryden M. Cooke J.. (2017). Reactive oxygen species (ROS) and wound healing: the functional role of ROS and emerging ROS-modulating technologies for augmentation of the healing process. Int. Wound J. 14, 89–96. doi: 10.1111/iwj.2017.14.issue-1 26688157 PMC7950185

[B34] Dwivedi D. Dwivedi M. Malviya S. Singh V. (2017). Evaluation of wound healing, anti-microbial and antioxidant potential of Pongamia pinnata in wistar rats. J. traditional complementary Med. 7, 79–85. doi: 10.1016/j.jtcme.2015.12.002 PMC5198820 28053891

[B35] Dwivedi S. Wahab R. Khan F. Mishra Y. K. Musarrat J. Al-Khedhairy A. A. (2014). Reactive oxygen species mediated bacterial biofilm inhibition via zinc oxide nanoparticles and their statistical determination. PloS One 9, e111289. doi: 10.1371/journal.pone.0111289 25402188 PMC4234364

[B36] El-Aassar M. Ibrahim O. M. Fouda M. M. El-Beheri N. G. Agwa M. M. (2020). Wound healing of nanofiber comprising Polygalacturonic/Hyaluronic acid embedded silver nanoparticles: *In-vitro* and *in-vivo* studies. Carbohydr Polym. 238, 116175. doi: 10.1016/j.carbpol.2020.116175 32299548

[B37] Elbialy Z. I. Atiba A. Abdelnaby A. Al-Hawary I. I. Elsheshtawy A. El-Serehy H. A.. (2020). Collagen extract obtained from Nile tilapia (Oreochromis niloticus L.) skin accelerates wound healing in rat model via up regulating VEGF, bFGF, and α-SMA genes expression. BMC Vet. Res. 16, 1–11. doi: 10.1186/s12917-020-02566-2 32972407 PMC7513287

[B38] El Gazaerly H. Elbardisey D. M. Eltokhy H. M. Teaama D. (2013). Effect of transforming growth factor Beta 1 on wound healing in induced diabetic rats. Int. J. Health Sci. 7, 160. doi: 10.12816/0006040 PMC3883606 24421745

[B39] El-Missiry M. A. Amer M. A. Hemieda F. A. Othman A. I. Sakr D. A. Abdulhadi H. L. (2015). Cardioameliorative effect of punicalagin against streptozotocin-induced apoptosis, redox imbalance, metabolic changes and inflammation. Egyptian J. Basic Appl. Sci. 2, 247–260. doi: 10.1016/j.ejbas.2015.09.004

[B40] Elzayat E. M. Auda S. H. Alanazi F. K. Al-Agamy M. H. (2018). Evaluation of wound healing activity of henna, pomegranate and myrrh herbal ointment blend. Saudi Pharm. J. 26, 733–738. doi: 10.1016/j.jsps.2018.02.016 29991918 PMC6035320

[B41] Firooz A. Nafisi S. Maibach H. I. (2015). Novel drug delivery strategies for improving econazole antifungal action. Int. J. Pharm. 495, 599–607. doi: 10.1016/j.ijpharm.2015.09.015 26383840

[B42] Gallagher K. A. Liu Z.-J. Xiao M. Chen H. Goldstein L. J. Buerk D. G.. (2007). Diabetic impairments in NO-mediated endothelial progenitor cell mobilization and homing are reversed by hyperoxia and SDF-1α. J. Clin. Invest. 117, 1249–1259. doi: 10.1172/JCI29710 17476357 PMC1857264

[B43] Gil J. Solis M. Higa A. Davis S. C. (2022). Candida albicans Infections: a novel porcine wound model to evaluate treatment efficacy. BMC Microbiol. 22, 1–9. doi: 10.1186/s12866-022-02460-x 35120444 PMC8815218

[B44] Gorup L. F. Longo E. Leite E. R. Camargo E. R. (2011). Moderating effect of ammonia on particle growth and stability of quasi-monodisperse silver nanoparticles synthesized by the Turkevich method. J. Colloid Interface Sci. 360 (2), 355–358. doi: 10.1016/j.jcis.2011.04.099 21616500

[B45] Hu Y. Xiong Y. Zhu Y. Zhou F. Liu X. Chen S.. (2023). Copper-epigallocatechin gallate enhances therapeutic effects of 3D-printed dermal scaffolds in mitigating diabetic wound scarring. ACS Appl. Materials Interfaces 15, 38230–38246. doi: 10.1021/acsami.3c04733 PMC10436249 37535406

[B46] Ismail T. Sestili P. Akhtar S. (2012). Pomegranate peel and fruit extracts: a review of potential anti-inflammatory and anti-infective effects. J. Ethnopharmacol 143, 397–405. doi: 10.1016/j.jep.2012.07.004 22820239

[B47] Ivashchenko O. Przysiecka Ł Peplińska B. Jarek M. Coy E. Jurga S. (2018). Gel with silver and ultrasmall iron oxide nanoparticles produced with Amanita muscaria extract: physicochemical characterization, microstructure analysis and anticancer properties. Sci. Rep. 8, 13260. doi: 10.1038/s41598-018-31686-x 30185987 PMC6125601

[B48] Jindam A. Yerra V. G. Kumar A. (2017). Nrf2: a promising trove for diabetic wound healing. Ann. Trans. Med. 5, 469. doi: 10.21037/atm.2017.09.03 PMC5733319 29285502

[B49] Joshi A. S. Singh P. Mijakovic I. (2020). Interactions of gold and silver nanoparticles with bacterial biofilms: Molecular interactions behind inhibition and resistance. Int. J. Mol. Sci. 21, 7658. doi: 10.3390/ijms21207658 33081366 PMC7589962

[B50] Karim S. Alkreathy H. M. Ahmad A. Khan M. I. (2021). Effects of methanolic extract based-gel from Saudi pomegranate peels with enhanced healing potential on excision wounds in diabetic rats. Front. Pharmacol. 12, 704503. doi: 10.3389/fphar.2021.704503 34122120 PMC8194859

[B51] Kataoka M. Minami K. Takagi T. Amidon G. E. Yamashita S. (2021). *In vitro*–*in vivo* correlation in cocrystal dissolution: consideration of drug release profiles based on coformer dissolution and absorption behavior. Mol. Pharm. 18, 4122–4130. doi: 10.1021/acs.molpharmaceut.1c00537 34618448

[B52] Kaur R. Mehan S. Khanna D. Kalra S. (2015). Polyphenol ellagic acid–targeting to brain: A hidden treasure. Int. J. Neurol. Res. 1, 141–152.

[B53] Kiritsi D. Nyström A. (2018). The role of TGFβ in wound healing pathologies. Mech. Ageing Dev. 172, 51–58. doi: 10.1016/j.mad.2017.11.004 29132871

[B54] Kurtzman C. P. Fell J. W. Boekhout T. Robert V. (2011). “Methods for isolation, phenotypic characterization and maintenance of yeasts,” in The yeasts (Amsterdam: Elsevier), 87–110.

[B55] Kushwaha A. Goswami L. Kim B. S. (2022). Nanomaterial-based therapy for wound healing. Nanomaterials 12, 618. doi: 10.3390/nano12040618 35214947 PMC8878029

[B56] Lara H. Romero-Urbina D. G. Pierce C. Lopez-Ribot J. L. Josefina M. (2015). Arellano-Jiménez and M Jose-Yacaman Effect of silver nanoparticles on Candida albicans biofilms: an ultrastructural study. J. Nanobiotechnol 13, 91. doi: 10.1186/s12951-015-0147-8 PMC4678641 26666378

[B57] Lázaro-Martínez J. L. Álvaro-Afonso F. J. Sevillano-Fernández D. Molines-Barroso R. J. García-Álvarez Y. García-Morales E. (2019). Clinical and antimicrobial efficacy of a silver foam dressing with silicone adhesive in diabetic foot ulcers with mild infection. Int. J. Lower Extremity Wounds 18, 269–278. doi: 10.1177/1534734619866610 31379224

[B58] Lee E. J. Lee J. H. Jin L. Jin O. S. Shin Y. C. Oh S. J.. (2014). Hyaluronic acid/poly (lactic-co-glycolic acid) core/shell fiber meshes loaded with epigallocatechin-3-O-gallate as skin tissue engineering scaffolds. J. Nanoscience Nanotechnology 14, 8458–8463. doi: 10.1166/jnn.2014.9922 25958546

[B59] Liu T. Han M. Tian F. Cun D. Rantanen J. Yang M. (2018). Budesonide nanocrystal-loaded hyaluronic acid microparticles for inhalation: in *vitro* and in *vivo* evaluation. Carbohydr Polym 181, 1143–1152. doi: 10.1016/j.carbpol.2017.11.018 29253943

[B60] Liu X. Lee Py Ho Cm Lui V. C. Chen Y. Che Cm. (2010). Silver nanoparticles mediate differential responses in keratinocytes and fibroblasts during skin wound healing. ChemMedChem 5, 468–475. doi: 10.1002/cmdc.200900502 20112331

[B61] Livak K. J. Schmittgen T. D. (2001). Analysis of relative gene expression data using real-time quantitative PCR and the 2– ΔΔCT method. Methods 25, 402–408. doi: 10.1006/meth.2001.1262 11846609

[B62] Loboda A. Damulewicz M. Pyza E. Jozkowicz A. Dulak J. (2016). Role of Nrf2/HO-1 system in development, oxidative stress response and diseases: an evolutionarily conserved mechanism. Cell Mol. Life Sci. 73, 3221–3247. doi: 10.1007/s00018-016-2223-0 27100828 PMC4967105

[B63] Long M. Rojo de la Vega M. Wen Q. Bharara M. Jiang T. Zhang R.. (2016). An essential role of NRF2 in diabetic wound healing. Diabetes 65, 780–793. doi: 10.2337/db15-0564 26718502 PMC4764153

[B64] Loreto F. Velikova V. (2001). Isoprene produced by leaves protects the photosynthetic apparatus against ozone damage, quenches ozone products, and reduces lipid peroxidation of cellular membranes. Plant Physiol. 127, 1781–1787. doi: 10.1104/pp.010497 11743121 PMC133581

[B65] Luengo J. Weiss B. Schneider M. Ehlers A. Stracke F. König K.. (2006). Influence of nanoencapsulation on human skin transport of flufenamic acid. Skin Pharmacol. Physiol. 19, 190–197. doi: 10.1159/000093114 16679821

[B66] Lukiswanto B. S. Miranti A. Sudjarwo S. A. Primarizky H. Yuniarti W. M. (2019). Evaluation of wound healing potential of pomegranate (Punica granatum) whole fruit extract on skin burn wound in rats (Rattus norvegicus). J. advanced veterinary Anim. Res. 6, 202. doi: 10.5455/javar.2019.f333 PMC6702873 31453192

[B67] Makvandi P. Ali G. W. Della Sala F. Abdel-Fattah W. I. Borzacchiello A. (2019). Biosynthesis and characterization of antibacterial thermosensitive hydrogels based on corn silk extract, hyaluronic acid and nanosilver for potential wound healing. Carbohydr Polym 223, 115023. doi: 10.1016/j.carbpol.2019.115023 31427021

[B68] Masoko P. Picard J. Howard R. Mampuru L. Eloff J. (2010). *In vivo* antifungal effect of Combretum and Terminalia species extracts on cutaneous wound healing in immunosuppressed rats. Pharm. Biol. 48, 621–632. doi: 10.3109/13880200903229080 20645734

[B69] Minghetti P. Cilurzo F. Casiraghi A. Montanari L. (2006). Evaluation of ex vivo human skin permeation of genistein and daidzein. Drug Delivery 13, 411–415. doi: 10.1080/10717540500466089 17002968

[B70] Nailis H. Kucharíková S. Řičicová M. Van Dijck P. Deforce D. Nelis H.. (2010). Real-time PCR expression profiling of genes encoding potential virulence factors in Candida albicans biofilms: identification of model-dependent and-independent gene expression. BMC Microbiol. 10, 1–11. doi: 10.1186/1471-2180-10-114 20398368 PMC2862034

[B71] Nayak S. B. Isik K. Marshall J. R. (2017). Wound-Healing potential of oil of Hypercium perforatum in excision wounds of male sprague dawley rats. Adv. Wound Care 6, 401–406. doi: 10.1089/wound.2017.0746 PMC5734146 29279803

[B72] Nirwana I. Munadziroh E. Yuliati A. Fadhila A. I. Wardhana A. S. Shariff K. A.. (2022). Ellagic acid and hydroxyapatite promote angiogenesis marker in bone defect. J. Oral. Biol. craniofacial Res. 12, 116–120. doi: 10.1016/j.jobcr.2021.11.008 PMC8605383 34840942

[B73] Okur M. E. Karantas I. D. Şenyiğit Z. Okur NÜ Siafaka P. I. (2020). Recent trends on wound management: New therapeutic choices based on polymeric carriers. Asian J. Pharm. Sci. 15, 661–684. doi: 10.1016/j.ajps.2019.11.008 33363624 PMC7750807

[B74] Olczyk P. Mencner Ł Komosinska-Vassev K. (2014). The role of the extracellular matrix components in cutaneous wound healing. BioMed. Res. Int 2014, 747584. doi: 10.1155/2014/747584 24772435 PMC3977088

[B75] Ortega S. Ittmann M. Tsang S. H. Ehrlich M. Basilico C. (1998). Neuronal defects and delayed wound healing in mice lacking fibroblast growth factor 2. Proc. Natl. Acad. Sci. 95, 5672–5677. doi: 10.1073/pnas.95.10.5672 9576942 PMC20437

[B76] Özkan M. Kırca A. Cemeroğlu B. (2004). Effects of hydrogen peroxide on the stability of ascorbic acid during storage in various fruit juices. Food Chem. 88, 591–597. doi: 10.1016/j.foodchem.2004.02.011

[B77] Paczkowska-Walendowska M. Ignacyk M. Miklaszewski A. Plech T. Karpiński T. M. Kwiatek J.. (2024). Electrospun nanofibers with pomegranate peel extract as a new concept for treating oral infections. Materials 17, 2558. doi: 10.3390/ma17112558 38893822 PMC11173823

[B78] Patra J. K. Baek K.-H. (2017). Antibacterial activity and synergistic antibacterial potential of biosynthesized silver nanoparticles against foodborne pathogenic bacteria along with its anticandidal and antioxidant effects. Front. Microbiol. 8, 227226. doi: 10.3389/fmicb.2017.00167 PMC5309230 28261161

[B79] Perween N. Khan H. Fatima N. (2019). Silver nanoparticles: an upcoming therapeutic agent for the resistant Candida infections. J. Microbiol. Exp. 7, 49–54. doi: 10.15406/jmen.2019.07.00240

[B80] Raina N. Rani R. Thakur V. K. Gupta M. (2023). New insights in topical drug delivery for skin disorders: from a nanotechnological perspective. ACS omega 8, 19145–19167. doi: 10.1021/acsomega.2c08016 37305231 PMC10249123

[B81] Ruffo M. Parisi O. I. Dattilo M. Patitucci F. Malivindi R. Pezzi V.. (2022). Synthesis and evaluation of wound healing properties of hydro-diab hydrogel loaded with green-synthetized AGNPS: *in vitro* and in ex vivo studies. Drug Delivery Transl. Res. 12 (8), 1881–1894. doi: 10.1007/s13346-022-01121-w PMC9242975 35359261

[B82] Sadeghian A. Ghorbani A. Mohamadi-Nejad A. Rakhshandeh H. (2011). Antimicrobial activity of aqueous and methanolic extracts of pomegranate fruit skin. Avicenna J. Phytomedicine 1, 67–73. doi: 10.22038/AJP.2011.123

[B83] Salama A. A. Ismael N. M. Bedewy M. (2021). The anti-inflammatory and antiatherogenic in *vivo* effects of pomegranate peel powder: from waste to medicinal food. J. Med. Food 24, 145–150. doi: 10.1089/jmf.2019.0269 32316851

[B84] Santos S. C. R. Miguel C. Domingues I. Calado A. Zhu Z. Wu Y.. (2007). VEGF and VEGFR-2 (KDR) internalization is required for endothelial recovery during wound healing. Exp. Cell Res. 313, 1561–1574. doi: 10.1016/j.yexcr.2007.02.020 17382929

[B85] Sayed S. Alotaibi S. S. El-Shehawi A. M. Hassan M. M. Shukry M. Alkafafy M.. (2022). The anti-inflammatory, anti-apoptotic, and antioxidant effects of a pomegranate-peel extract against acrylamide-induced hepatotoxicity in rats. Life 12, 224. doi: 10.3390/life12020224 35207511 PMC8878900

[B86] Scappaticci R. A. F. Berretta A. A. Torres E. C. Buszinski A. F. M. Fernandes G. L. Dos Reis T. F.. (2021). Green and chemical silver nanoparticles and pomegranate formulations to heal infected wounds in diabetic rats. Antibiotics 10, 1343. doi: 10.3390/antibiotics10111343 34827281 PMC8614779

[B87] Scheller J. Chalaris A. Schmidt-Arras D. Rose-John S. (2011). The pro-and anti-inflammatory properties of the cytokine interleukin-6. Biochim. Biophys. Acta (BBA)-Molecular Cell Res. 1813, 878–888. doi: 10.1016/j.bbamcr.2011.01.034 21296109

[B88] Seeram N. P. Zhang Y. Reed J. D. Krueger C. G. Vaya J. (2006). “Pomegranate phytochemicals,” in Pomegranates (Cambridge: CRC Press), 21–48.

[B89] Shafique M. Khan M. A. Khan W. S. Ahmad W. Khan S. (2017). Fabrication, characterization, and in *vivo* evaluation of famotidine loaded solid lipid nanoparticles for boosting oral bioavailability. J. Nanomaterials 2017, 7357150. doi: 10.1155/2017/7357150

[B90] Shalumon K. Sheu C. Chen C.-H. Chen S.-H. Jose G. Kuo C.-Y.. (2018). Multi-functional electrospun antibacterial core-shell nanofibrous membranes for prolonged prevention of post-surgical tendon adhesion and inflammation. Acta Biomater 72, 121–136. doi: 10.1016/j.actbio.2018.03.044 29626695

[B91] Shen H.-M. Chen C. Jiang J.-Y. Zheng Y.-L. Cai W.-F. Wang B.. (2017). The N-butyl alcohol extract from Hibiscus rosa-sinensis L. flowers enhances healing potential on rat excisional wounds. J. Ethnopharmacol 198, 291–301. doi: 10.1016/j.jep.2017.01.016 28088494

[B92] Simonsen L. Petersen M. B. Groth L. (2002). *In vivo* skin penetration of salicylic compounds in hairless rats. Eur. J. Pharm. Sci. 17, 95–104. doi: 10.1016/S0928-0987(02)00147-1 12356424

[B93] Skłodowski K. Chmielewska-Deptuła S. J. Piktel E. Wolak P. Wollny T. Bucki R. (2023). Metallic nanosystems in the development of antimicrobial strategies with high antimicrobial activity and high biocompatibility. Int. J. Mol. Sci. 24, 2104. doi: 10.3390/ijms24032104 36768426 PMC9917064

[B94] Sreekumar S. Sithul H. Muraleedharan P. Azeez J. M. Sreeharshan S. (2014). Pomegranate fruit as a rich source of biologically active compounds. BioMed. Res. Int. 2014, 686921. doi: 10.1155/2014/686921 24818149 PMC4000966

[B95] Taff H. T. Mitchell K. F. Edward J. A. Andes D. R. (2013). Mechanisms of Candida biofilm drug resistance. Future Microbiol. 8, 1325–1337. doi: 10.2217/fmb.13.101 24059922 PMC3859465

[B96] Tarusha L. Paoletti S. Travan A. Marsich E. (2018). Alginate membranes loaded with hyaluronic acid and silver nanoparticles to foster tissue healing and to control bacterial contamination of non-healing wounds. J. Mater Sci. Mater Med. 29, 1–14. doi: 10.1007/s10856-018-6027-7 29396683

[B97] Tayel A. A. El-Tras W. F. (2010). Anticandidal activity of pomegranate peel extract aerosol as an applicable sanitizing method. Mycoses 53, 117–122. doi: 10.1111/j.1439-0507.2008.01681.x 19207830

[B98] Tsang P. W.-K. Bandara H. Fong W.-P. (2012). Purpurin suppresses Candida albicans biofilm formation and hyphal development. PloS One 7, e50866. doi: 10.1371/journal.pone.0050866 23226409 PMC3511323

[B99] Um I. C. Fang D. Hsiao B. S. Okamoto A. Chu B. (2004). Electro-spinning and electro-blowing of hyaluronic acid. Biomacromolecules 5, 1428–1436. doi: 10.1021/bm034539b 15244461

[B100] Urciuolo F. Casale C. Imparato G. Netti P. A. (2019). Bioengineered skin substitutes: the role of extracellular matrix and vascularization in the healing of deep wounds. J. Clin. Med. 8, 2083. doi: 10.3390/jcm8122083 31805652 PMC6947552

[B101] Vazquez-Muñoz R. Avalos-Borja M. Castro-Longoria E. (2014). Ultrastructural analysis of Candida albicans when exposed to silver nanoparticles. PloS One 9, e108876. doi: 10.1371/journal.pone.0108876 25290909 PMC4188582

[B102] Venusova E. Kolesarova A. Horky P. Slama P. (2021). Physiological and immune functions of punicalagin. Nutrients 13, 2150. doi: 10.3390/nu13072150 34201484 PMC8308219

[B103] Wang P. H. Huang B.-S. Horng H.-C. Yeh C.-C. Chen Y.-J. (2018). Wound healing. J. Chin. Med. Assoc. 2, 94–101. doi: 10.1016/j.jcma.2017.11.002 29169897

[B104] Wang Z. Ou X. Guan L. Li X. Liu A. Li L.. (2023). Pomegranate-inspired multifunctional nanocomposite wound dressing for intelligent self-monitoring and promoting diabetic wound healing. Biosens Bioelectron 235, 115386. doi: 10.1016/j.bios.2023.115386 37187060

[B105] Witte M. B. Barbul A. (2002). Role of nitric oxide in wound repair. Am. J. Surg. 183, 406–412. doi: 10.1016/s0002-9610(02)00815-2 11975928

[B106] Yeligar S. M. Machida K. Kalra V. K. (2010). Ethanol-induced HO-1 and NQO1 are differentially regulated by HIF-1α and Nrf2 to attenuate inflammatory cytokine expression. J. Biol. Chem. 285, 35359–35373. 20833713 10.1074/jbc.M110.138636 PMC2975160

[B107] Yuniarti W. M. Primarizky H. Lukiswanto B. S. (2018). The activity of pomegranate extract standardized 40% ellagic acid during the healing process of incision wounds in albino rats (Rattus norvegicus). Veterinary world. 11, 321. 29657424 10.14202/vetworld.2018.321-326 PMC5891847

[B108] Zhang Y. Liu Y.-C. Chen S.-M. Jiang Y.-Y. An M.-M. (2021). Evaluation of the in *vitro* activity and in *vivo* efficacy of anidulafungin-loaded human serum albumin nanoparticles against Candida albicans. Front. Microbiol. 12, 788442. doi: 10.3389/fmicb.2021.788442 34970244 PMC8712755

[B109] Zhao Q. Xu J. Cheng Z. (2023). Growth differentiation factor 10 induces angiogenesis to promote wound healing in rats with diabetic foot ulcers by activating TGF-β1/Smad3 signaling pathway. Front. Endocrinol. (Lausanne) 13, 1013018. doi: 10.3389/fendo.2022.1013018 36714584 PMC9880151

[B110] Zhou J. Ni M. Liu X. Ren Z. Zheng Z. (2017). Curcumol promotes vascular endothelial growth factor (VEGF)-mediated diabetic wound healing in streptozotocin-induced hyperglycemic rats. Med. Sci. monitor: Int. Med. J. Exp. Clin. Res. 23, 555. doi: 10.12659/MSM.902859 PMC5297326 28138126

